# Janus CoMOF‐SEBS Membrane for Bifunctional Dielectric Layer in Triboelectric Nanogenerators

**DOI:** 10.1002/advs.202307656

**Published:** 2024-01-29

**Authors:** Hyunjoon Yoo, Manmatha Mahato, Ji‐Seok Kim, Saewoong Oh, Mousumi Garai, Van Hiep Nguyen, Ashhad Kamal Taseer, Myung‐Joon Lee, Il‐Kwon Oh

**Affiliations:** ^1^ National Creative Research Initiative for Functionally Antagonistic Nano‐Engineering Department of Mechanical Engineering Korea Advanced Institute of Science and Technology (KAIST) 291 Daehak‐ro Yuseong‐gu Daejeon 34141 Republic of Korea

**Keywords:** bifunctionality, gravitational sedimentation, metal–organic frameworks, self‐rehabilitation, stretchable TENG

## Abstract

Considerable research has been conducted on the application of functional nano‐fillers to enhance the power generation capabilities of triboelectric nanogenerators (TENGs). However, these additives often exhibit a decrease in output power at higher concentration. Here, a Janus cobalt metal–organic framework‐SEBS (JCMS) membrane is reported as a dual‐purpose dielectric layer capable of efficiently capturing and blocking charges for high‐performance TENGs. The JCMS is produced asymmetrically through gravitational sedimentation, employing spherical CoMOFs within a diluted SEBS solution. Beyond its dual dielectric characteristics, the JCMS showcases exceptional mechanical durability, displaying notable stretchability of up to 475% and remarkable resilience when subjected to diverse mechanical pressures. Consequently, the JCMS‐TENG produces a maximum peak‐to‐peak voltage of 936 V, a current of 42.8 µA, and a power density of 10.89 W m^−^^2^ when exposed to an external force of 10 N at a 5 Hz frequency. This investigation highlights the potential of JCMS‐TENGs with unique structures, known for their exceptional energy harvesting capabilities, mechanical strength, and flexibility. Additionally, the promising prospects of easily produced asymmetric structures is emphasized with bifunctionalities for developing efficient and flexible MOFs‐based TENGs. These advancements are well‐suited for self‐powered wearables, rehabilitation devices, and energy harvesters.

## Graphical abstract

Fabricated through a simple process that utilizes gravitational sedimentation, Janus cobalt metal–organic framework‐SEBS (JCMS) naturally forms a bifunctional layer, effectively trapping and blocking charges. This overcomes the limitations of conventional high‐loading composite‐based triboelectric nanogenerators devices. Combined with its high stretchability, mechanical robustness, and high performance, JCMS presents significant potential for future advancements in energy harvesting devices and self‐powered wearable sensor applications.

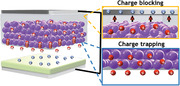

## 1 Introduction

Advancements in manufacturing technologies^[^ ^1^ ^]^ and soft electronics^[^ ^2^, ^3^, ^4^ ^]^ have led to rapid improvements in wearable electronic devices, expanding their potential for practical applications such as smart healthcare,^[^ ^5^ ^]^ the Internet of Things,^[^ ^6^ ^]^ and personalized rehabilitation ^[^ ^7^, ^8^ ^]^. One of the crucial aspects to make these devices a reality is ensuring user comfort during activities of daily living. However, realizing these devices with flexibility,^[^ ^9^ ^]^ stretchability,^[^ ^10^ ^]^ and sustainability^[^ ^6^ ^]^ remains challenging, primarily due to limitations in powering technologies. To overcome these limitations, triboelectric nanogenerators (TENGs) are emerging as an alternative power source.^[^ ^11^, ^12^ ^]^ This energy harvesting technology can easily convert mechanical energy into electrical energy through the combined effects of triboelectrification and electrostatic induction. With their high efficiency, simplicity in design, and adaptability, TENGs permit the utilization of flexible materials, such as foams,^[^ ^13^ ^]^ films,^[^ ^14^ ^]^ and cloths,^[^ ^15^ ^]^ consequently showing great potential as self‐powered active sensors and wearable device activation.

Efforts are currently underway to enhance the performance of wearable TENGs by modifying their constituent structural features and materials^[^ ^16^, ^17^, ^18^ ^]^ to meet the power requirements of wearable devices. In particular, nanocomposite‐based TENGs show significant promise due to their potential compatibility with wearable devices and their capacity for incorporating novel materials.^[^ ^19^, ^20^, ^21^, ^22^, ^23^ ^]^ These functional fillers can exchange charge between the friction surface and the filler via contact electrification (CE) and trap the exchanged electrons to increase the surface charge density. However, a significant obstacle in utilizing the filler arises as these composites often exhibit considerable degradation with an increased mass ratio to the matrix. The conventional method of mixing and casting composite at high mass ratios generally results in particle aggregation or agglomeration. This leads to both charge loss and mechanical weakness, ultimately reducing the performance of the TENGs.^[^ ^24^, ^25^ ^]^ This charge loss is mainly attributed to recombination of charges between the filler and counter material, or filler and electrode, resulting from exceeding percolation threshold of the filler network.^[^ ^26^, ^27^ ^]^ To prevent these recombination issues, the general strategy is to create a separate charge trap layer between electrode and tribo‐layer. However, this approach introduces manufacturing complications, often leading to delaminations or cracks during the stretching operation. Therefore, to fully leverage the potential of the filler material, it is more desirable to fabricate multifunctional tribo‐layers within a single fabrication step.

Interestingly, there was a successful attempt to fabricate electrodes by a one‐pot fabrication using gravity. This fabrication method utilizes gravitational sedimentation, which is a natural phenomenon where heavier suspended particles separate from the solvent due to gravity. While there are only few articles for forming membranes by gravitational sedimentation, previous research in TENG field has successfully produced stretchable electrodes using gallium metal and CNTs.^[^ ^28^, ^29^ ^]^ They utilized the force of gravity to produce an asymmetric structure, known as the Janus structure, enabling the formation of structures with different properties (i.e., charge generating and collecting for versatile applications). Inspired by these findings, we aim to create a charge‐controllable Janus dielectric layer with high mass ratio using the gravitational sedimentation method. It is believed that if bifunctionality for charge trapping and charge blocking is naturally established, it could potentially minimize charge loss due to recombination. To the best of our knowledge, no Janus composite film has been fabricated to enhance the performance of TENGs using gravitational sedimentation till date.

In this study, we report a Janus cobalt metal–organic framework (CoMOF)‐styrene‐ethylene‐butylene‐styrene (SEBS) membrane (JCMS) with bifunctional dielectric properties that can both effectively trap and block charges in TENGs. The asymmetric structure was fabricated by inducing gravitational sedimentation of CoMOFs in a SEBS‐toluene solution. Upon drying, a charge‐blocking layer containing only SEBS forms on the top of the charge‐trapping layer of the CoMOFs membrane. This asymmetric structure of the JCMS exhibits excellent flexibility and stretchability, as well as a bifunctionality to serve as both blocking and trapping charges. These characteristics make JCMS a promising candidate for the development of future high‐powered, wearable TENGs. The dense layer of CoMOFs in the JCMS was utilized as a tribopositive contact surface in the fabrication of TENG devices (JCMS‐TENG). The effect of the mass ratio on the charge blocking and charge trapping performances was systematically explored through morphological analysis and electrical characterization of the JCMS‐TENG. The JCMS‐TENG demonstrated a 327% enhancement in output voltage at an optimized mass ratio of 50 wt.% compared to the conventional SEBS, indicating a substantially higher improvement than those reported in prior studies on the filler content of the Janus structure (Table S1, Supporting Information). The effects of applied force and contact‐separation frequency on the output performance of TENGs were also investigated. Furthermore, the practical applicability of the TENGs was demonstrated through the implementation of a finger movement sensor for rehabilitation. This study suggests that the JCMS, fabricated through a simple one‐step process, has the potential to be adopted in high‐performance, flexible, and stretchable energy harvesting systems and self‐powered wearable devices, and active sensors.

## 2 Results and Discussion

**Figure 1:**
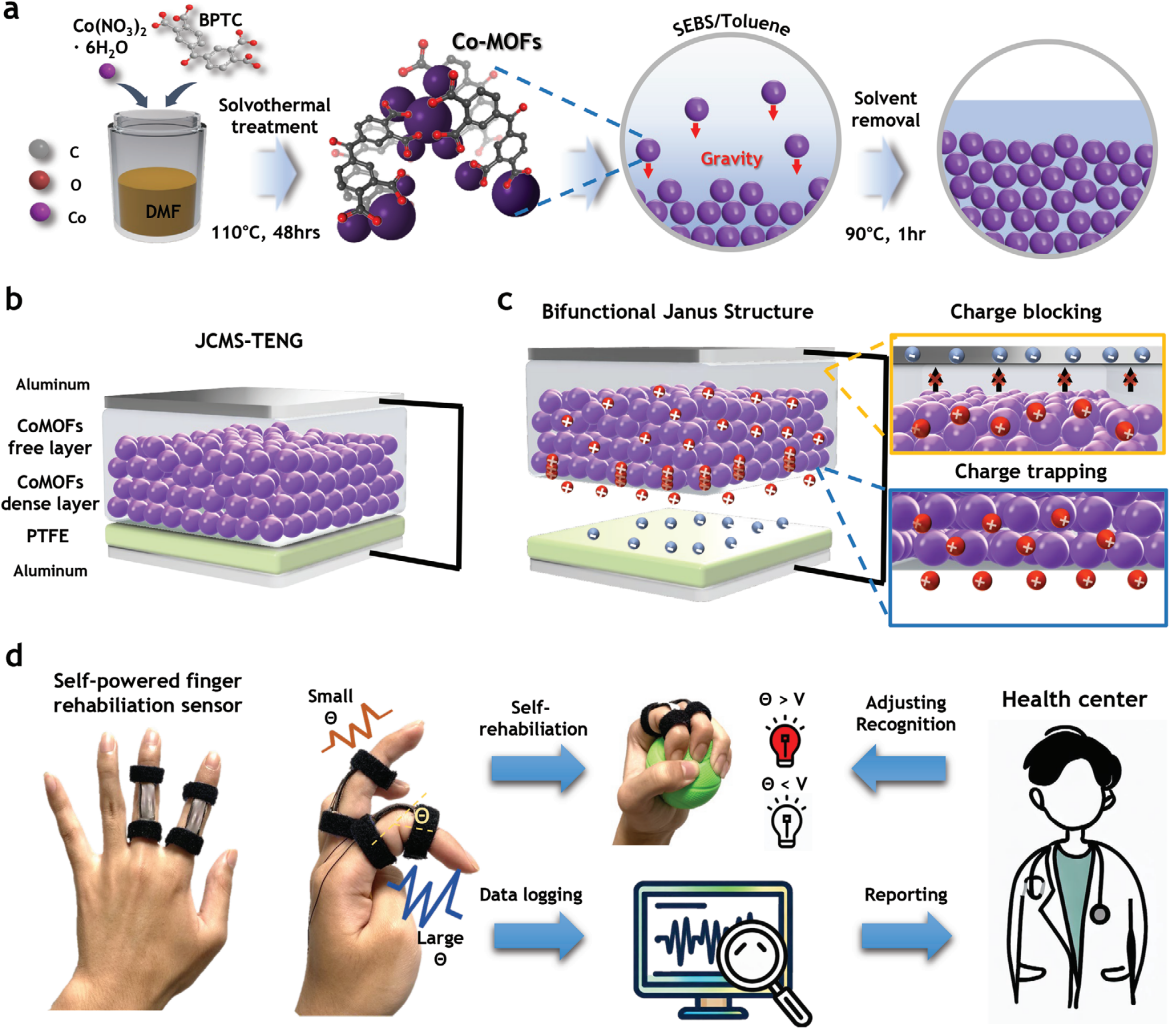
CoMOF‐based TENG prepared by gravitational sedimentation method. a) Schematic diagram illustrating the preparation of JCMS through gravitational sedimentation. b) Schematic of the JCMS‐TENG device. c) Illustration explaining the function of charge blocking and charge trapping layer resulting from the asymmetric structure of the JCMS. d) Schematic diagram of wJCMS‐TENG‐based stretchable self‐powered finger rehabilitation sensor and its application to smart healthcare.

While a variety of materials are being investigated as filler for charge trapping, we introduce a new species of CoMOFs and their usage in designing bifunctional asymmetric structure. **Figure** **1**a illustrates the process of obtaining CoMOFs and their asymmetric membrane. The CoMOFs are synthesized by solvothermal treatment of cobalt(II) nitrate hexahydrate and BPTC. To induce a homogeneous suspension, these CoMOFs particles are pre‐dispersed in 1,4 dioxane and then mixed with the dispersion of SEBS in toluene, as described in Figure S1 (Supporting Information). Once this mixed solution is poured into a glass mold, gravitational sedimentation occurs due to the density difference between the solution and the CoMOFs particles. The settling velocity can be described by Equation 1, known as Stoke's law.

(1) $$V=\frac{2\left(\rho_{s}-\rho_{f}\right)g\alpha^{2}}{9\eta }$$

where *V* is the settling velocity, η is the viscosity of fluid, ρ_*s*_ is the density of particle, α is the radius of particle, *g* is the acceleration of gravity, and ρ_*f*_ is the density of suspension. To reduce fabrication time, a sufficiently dilute concentration of polymer solution was used to decrease viscosity. The detailed fabrication method is described in the experimental section. Through gravitational sedimentation, the JCMS membrane is divided into two regions; particle‐dense and particle‐free (i.e., only SEBS polymer) regions, as shown in Figure 1b. At the particle‐dense region, the SEBS networks are formed between micro‐sized particles, which not only can hold the particles, but also can be mechanically deformed due to intrinsic elastic behavior of SEBS^[^ ^30^ ^]^ (Figure 1c). In addition, the CoMOFs, which densely form within the SEBS matrix due to gravity, will trap and drive more charge into this region during triboelectrification. However, charges can also flow between the particles in the CoMOFs dense region due to contact with each other, which is considered as a defect that causes leakage current in other studies.^[^ ^31^, ^32^, ^33^ ^]^ In general, once the charges start to flow through the filler material, these will flow to the electrodes attached to the dielectric layer, eventually leading to performance degradation. Here, the additional SEBS layer obtained naturally through sedimentation contains styrene inside the chain, which is well‐known as charge blocking material^[^ ^34^ ^]^ preventing the recombination with the electron inside electrode. By utilizing the properties of this unique structure, we have fabricated an all‐stretchable TENG by utilizing JCMS with wrinkled electrode (wJCMS) that can be applied to flexible, stretchable self‐powered sensors for finger rehabilitation (Figure 1d). This stretchable feature of the sensor allows it to sense the angle of the finger without causing discomfort to the user. Furthermore, the sensor facilitates the implementation of smart healthcare, allowing users to transmit data to a health center from any location. The experts in the health center can provide feedback and guidance to patients using data to facilitate efficient rehabilitation towards a specific target angle, without in‐person visits.

**Figure 2:**
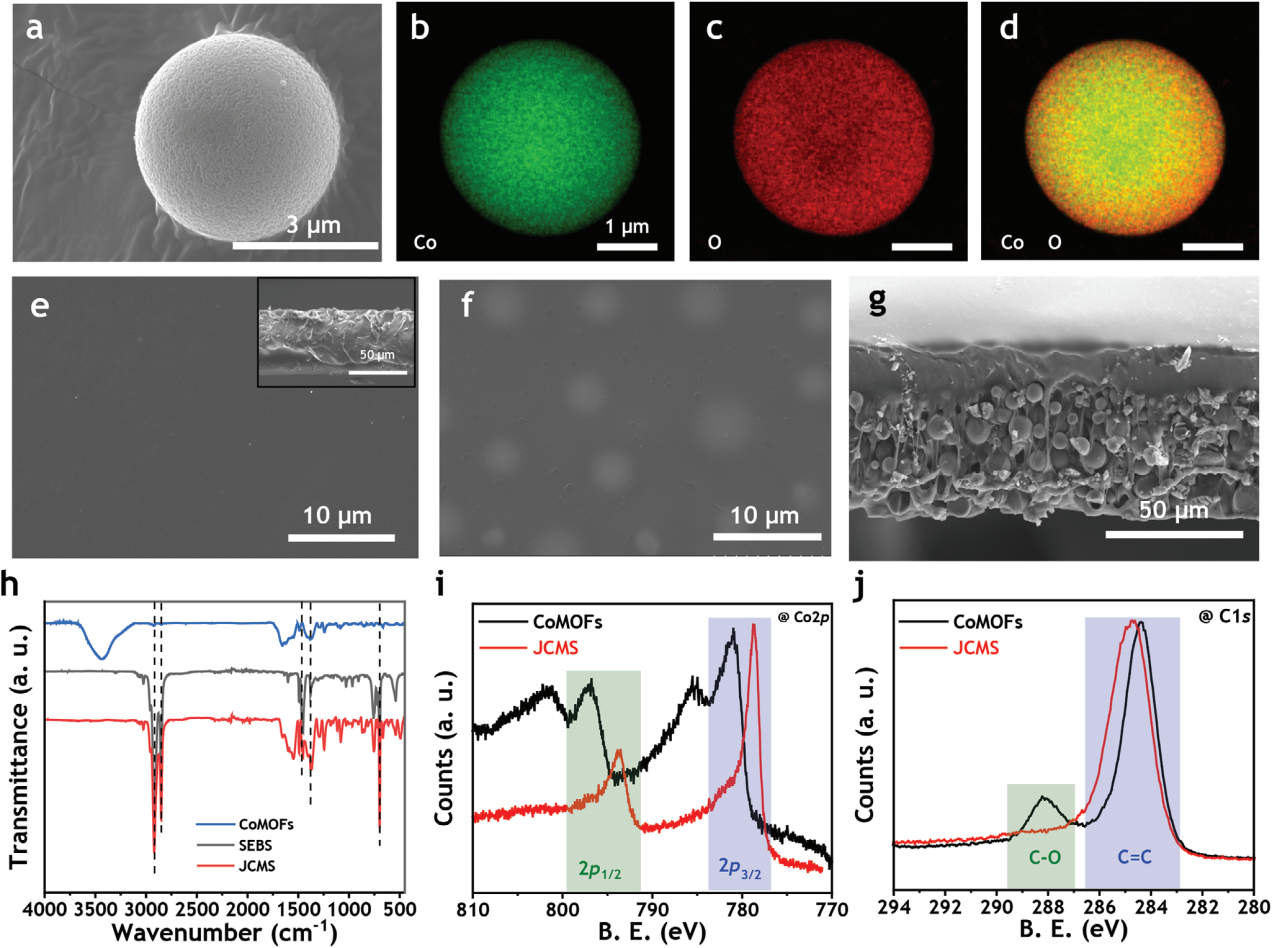
Characterization of CoMOFs and JCMS. a) SEM image of a CoMOFs particle displaying spherical structure following solvothermal treatment. b–d) Elemental images of a CoMOFs particle, corresponding (b) cobalt, (c) oxygen, and (d) their overlapped, displaying clear presence of each element. e–g) Morphological characterization of pure SEBS and 50 wt.% of JCMS. The SEM image of surface morphology shows CoMOFs (f), in contrast to pure SEBS (e). The cross‐sectional image shows the formation of an asymmetric structure with free and dense CoMOFs layers due to the effects of gravitational sedimentation (g), distinguishable from the inset of (e). h) FTIR‐ATR spectra of SEBS, CoMOFs, and JCMS. i,j) XPS spectra of CoMOFs and JCMS centered on (i) Co 2p and (j) C 1s.

To investigate the surface morphology and chemical composition of the synthesized CoMOFs and JCMS, field‐emission scanning electron microscopy (FE‐SEM) and transmission electron microscopy (TEM) were conducted, as shown in **Figure** **2**a–g. The micro‐sized spherical particles, with diameters ranging from 1 to 10 µm, were grown into a dense manner, as depicted in Figure 2a and Figure S2 (Supporting Information). The natural sedimentation is mainly influenced by gravity and Brownian diffusion. For submicron suspensions, the Brownian diffusion can induce random walk characteristic, leading to sparse formation of deposited layer.^[^ ^35^ ^]^ Hence, it is desirable to utilize the micro‐sized particles not only to shorten the fabrication time, but also to avoid too loosely packed structure. The energy dispersive X‐ray spectroscopy (EDS) mapping (Figure 2b–d) showed that each element was uniformly distributed in the particles, indicating that the cobalt and organic ligands used as precursors were successfully coordinated. Next, we compare the surface morphology and cross‐section of JCMS and pure SEBS, prepared by gravitational sedimentation (Figure 2e–g). Compared to the pure SEBS (Figure 2e), the presence of CoMOFs on the contact surface is noticeable. (Figure 2f). Also, the dense CoMOFs region and neat SEBS layer are clearly distinguished in the cross‐sectional image of JCMS in Figure 2g. For the understanding of the effective electronic interaction between SEBS and CoMOFs, a series of spectroscopic characterizations have been performed (Figure 2h–j; Figure S3, Supporting Information). To confirm the presence of typical chemical functional groups of SEBS, CoMOFs, and JCMS, fourier‐transform infrared spectroscopy (FT‐IR) analysis was performed and shown in Figure 2h. It was confirmed that JCMS consists of all the characteristic transmission peaks associated with the SEBS and CoMOFs. The powder XRD pattern shows semi‐crystalline phase of Co‐MOFs. The spectrum displays a narrow XRD peak at 2θ of 6.9° with an additional broad peak at 20.2° (Figure S4a, Supporting Information). The appearances of characteristic Raman shift evidently support the structural configuration of Co‐MOFs. The expected chemical functionalities were well reflected in the Raman spectrum, as shown in Figure S4b (Supporting Information).^[^ ^36^ ^]^ X‐ray photoelectron spectroscopy analysis shows that the π‐electrons cloud of SEBS interacts significantly with the Co‐metal center by systematically shifting the binding energies toward lower values for Co2p electronic configurations. As seen in Figure 2i, the binding energy values of Co2p_3/2_ (780.98 eV) and Co2p_1/2_ (796.83 eV) for pure CoMOFs were dropped down to 778.73 and 793.68 eV, respectively in the JCMS. The C1s XPS spectra corresponding to C═C and C─O functional moieties (Figure 2j) and O1s spectra (Figure S3, Supporting Information) of CoMOFs were also shifted toward relatively higher binding energies, confirming that there is a strong physicochemical interaction between CoMOFs and SEBS. As a result, a stable state of the JCMS structure was obtained, where a well‐ordered distribution of MOFs particles was maintained on the bottom surfaces of SEBS matrices. To confirm the surface porosity and polarity, the physisorption study of CoMOFs was carried out under carbon dioxide environment at 273 K (Figure S5, Supporting Information). The CoMOFs was found to have a specific BET surface area of 198.4 m^2^g^−1^. Also, a CO_2_ uptake capacity of 1.9 mmolg^−1^ is observed at a relatively low pressure (up to 1 bar) at 273 K. signifying the effective surface polarities available for interaction with guest materials.

**Figure 3:**
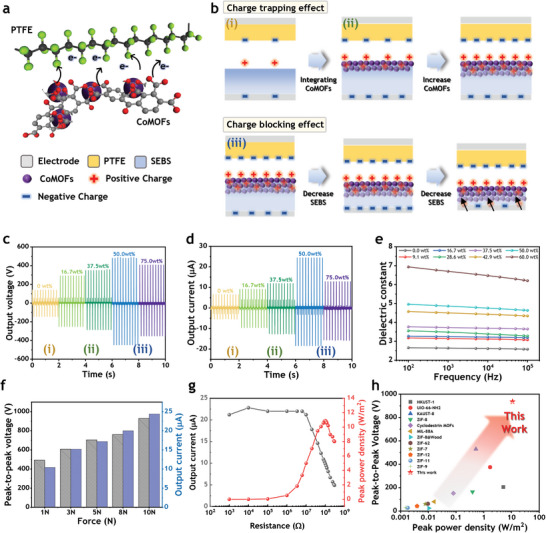
Triboelectric performance of JCMS. a) Illustration of the proposed mechanism for triboelectric effects resulting from contact between CoMOFs dense layer and PTFE. b) Illustration of the mechanism for each layer of JCMS based on mass ratio. (i) Pure SEBS‐TENG without CoMOFs (ii) By controlling the quantity of CoMOFs, charge trapping effect is observed through the increased CoMOFs dense layer (iii) By controlling the quantity of SEBS, the charge blocking effect is observed through the reduced pure SEBS layer. c,d) Output performance of voltage (c) and current (d) for different mass ratio. e) Dielectric constant for different mass ratio. f) Output voltage of the optimized JCMS‐TENG under various external forces. g) Output current and peak power density for various load resistance. h) Comparative plot of peak‐to‐peak voltage and peak power density with other MOF‐based TENGs.

To investigate the influence of the asymmetric structure of the JCMS on the output performance of the TENG, the electrical characterization of the contact‐separation mode TENG was conducted (**Figure** **3**). For maximizing the triboelectrification effect of the TENG, polytetrafluoroethylene (PTFE) was selected as the tribonegative material because of its high electron affinity, while the JCMS membrane was used as the tribopositive material. The possible tribo‐interaction between the most electronegative fluorines and the electropositive oxygens is shown schematically in Figure 3a. It is believed that Co on CoMOFs enriches the density of oxygen atoms surrounding it and significantly increases the effective electronic interaction during the friction with the fluorines of PTFE. To examine the mechanisms behind the asymmetric structure, three different sample ranges were used depending on mass ratio, as illustrated in Figure 3b: i) Pure SEBS, ii) a controlled absolute mass of CoMOFs (20 mg to 120 mg) with a fixed quantity of SEBS (200 mg) to verify the charge trapping effect of CoMOFs particles, and iii) a controlled absolute mass of SEBS (200 to 40 mg) with a fixed quantity of CoMOFs (120 mg) to verify the charge blocking effect of the SEBS (Table S2, Supporting Information). The cross‐sectional SEM images of each JCMS are displayed in Figure S6 (Supporting Information). As the mass ratio changed from 9.1 to 37.5 wt.%, the thickness of dense CoMOFs region gradually increased, and from 42.9 to 75 wt.%, the thickness of pure SEBS layer gradually decreased with a higher mass ratio, while the dense CoMOFs layer retained its structure. This phenomenon is attributed to the fact that the dense CoMOFs layer formed first, followed by the formation of the pure SEBS layer during evaporation. First, to verify the charge trapping effect of CoMOF particles, contact‐separation tests were performed for mass ratios ranging from 9.1 to 37.5 wt.%. The test is conducted using a force of 10 N, and at a frequency of 5 Hz. With the increase of mass ratio, the output performance significantly increased from peak‐to‐peak voltage of 286 V, and output current of 6.56 µA to 636 V, 12.7 µA (Figure 3c,d), with respect to thickened CoMOFs layers. The overall performances including output charge are displayed in Figure S7 (Supporting Information), with respect to mass ratio. This enhancement suggests that CoMOFs can trap more amount of charge than those they lost to environment, and they can continuously accumulate the charge inside the structure, even when there is charge flow within the aggregated state. Unlike other studies embedding fillers with high mass ratio, we note that the SEBS layer can block the recombination between filler and electrode area, leading to continuous enhancement of output performance. However, excessive thickness of charge trapping layer may hinder the electrostatic induction on the electrode attached to the JCMS due to the degradation of electrostatic field generated via CE. Therefore, the optimization process was carried by controlling the SEBS amount (i.e., mass ratio of 42.9 to 75 wt.%). The JCMS‐TENG exhibited peak‐to‐peak output voltage of 936 V and output current of 42.8 µA, making a 327% and 357% increase in output voltage and current, respectively, compared to the pure SEBS. The JCMS‐TENG was optimized at mass ratio of 50 wt.% for better TENG performance. Above the optimization point, the thickness of the SEBS layer is significantly thin (Figure S6g,h, Supporting Information), leading to the performance drop due to leakage current between fillers and electrode. To investigate charge transfer between PTFE and JCMS, surface potential measurements were conducted. Figure S8 (Supporting Information) shows the difference in surface potential before and after sufficient contact‐separation of PTFE with JCMS having different mass ratios. The surface potential of JCMS exhibited a positive value upon contact with PTFE. The maximum difference in surface potential was observed with a 50 wt.% CoMOFs loading. The results suggest that the charge trapping capacity of JCMS increases with the increasing proportion of CoMOFs, a charge trapping material, as illustrated in Figure 3b. However, after 60 wt.%, the difference in surface potential starts to decrease. This is a result of the decreased charge capacity that JCMS can store due to leakage currents occurring at the electrode caused by the relatively thinner SEBS.

To further investigate the electrical influences on output performance, a dielectric constant measurement was conducted for each mass ratio of the JCMS. The relationship between dielectric constant of material and charge density of electrode $\sigma_{o}^{\prime }$, is expressed in Equation (2):

(2)

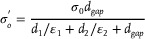

where σ_0_ is the triboelectric charge density; ε_1_ and ε_2_ are the dielectric constant of PTFE and JCMS, respectively; *d*_1_, *d*_2_, and *d_gap_* represent the thickness of PTFE, JCMS, and the gap distance between PTFE and JCMS, respectively. Equation (2) demonstrates that an increased ratio of dielectric material (i.e., triboelectric layer) induces more charges in the electrode. The dielectric constant of 2.6 was obtained for the pure SEBS, aligning with a previous study^[^ ^37^ ^]^ (Figure 3e). As the mass ratio increased, the dielectric constant elevated from 2.64 (for pure SEBS) to 6.70 (for a mass ratio of 60 wt.%) at a frequency of 1 kHz. These trends are consistent with the charge output (Figure S7c, Supporting Information) up to optimized mass ratio. The enhancement of charge is due to combined effect of charge trapping and charge blocking effect of JCMS, as well as enhanced dielectric constant. However, for mass ratio over 60 wt.%, the charges start to decrease even though the dielectric constant continues to increase.

The correlation between the dielectric constant and TENG performance can be influenced by factors such as surface roughness and leakage current. To address surface roughness, the reduced effective contact area may cause discrepancies between the trends in dielectric constant and TENG performance.^[^ ^31^, ^38^ ^]^

**Figure 4:**
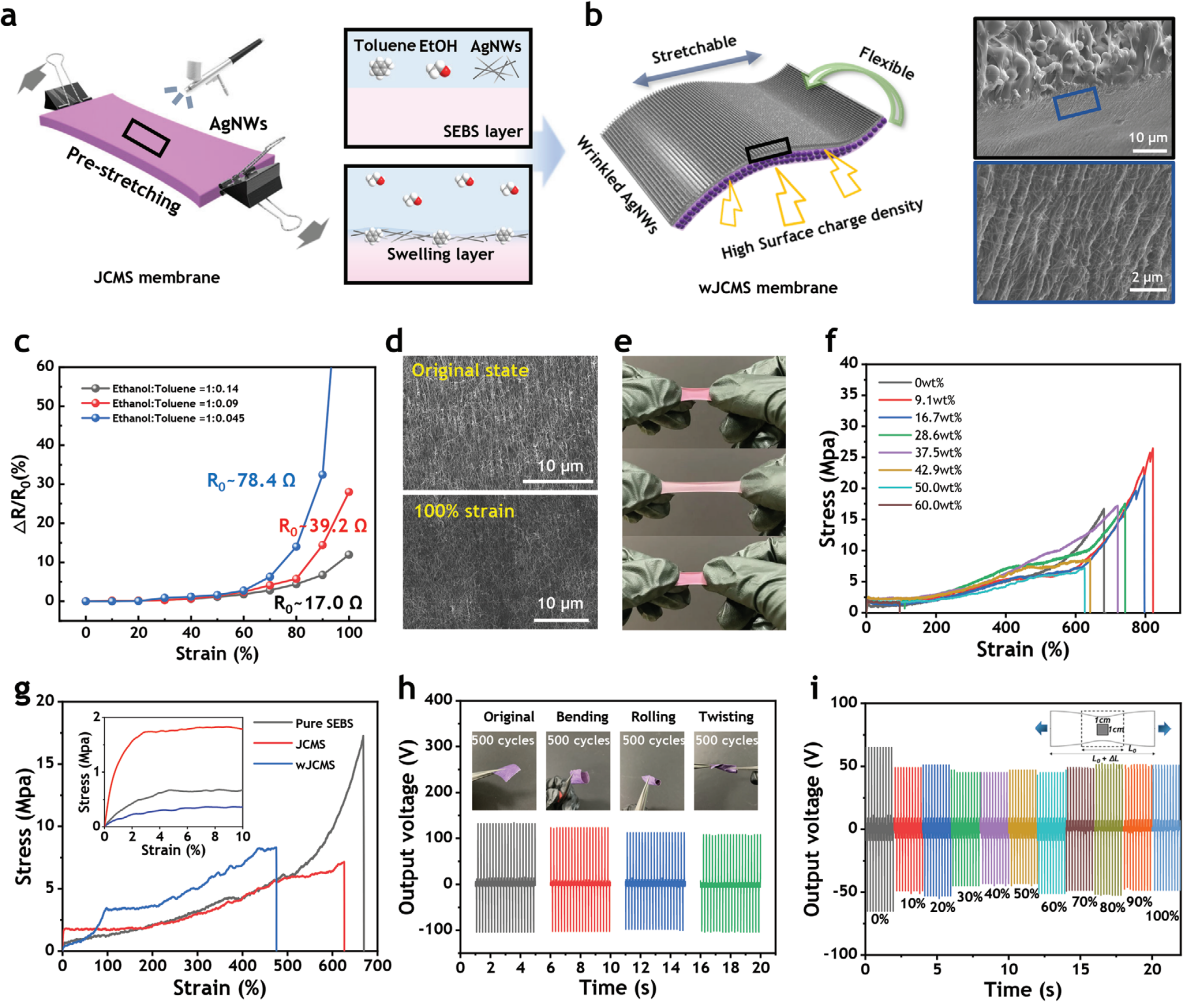
Mechanical/Triboelectrical Properties of JCMS‐TENGs as Wearable TENGs. a) Schematic illustration for fabricating wrinkled silver nanowire electrodes via surface dissolution effect. b) The fabricated stretchable, flexible wJCMS. The SEM image on the right shows the silver nanowires partially embedded in the SEBS surface. c) Relative change of resistance versus strain with different ratio of toluene and ethanol for spray coating method. d) The SEM images showing wrinkle morphology before and after 100% strain. e) A digital photograph of JCMS showing the reversible stretchability over 300% strain. f) Stress–strain curves of JCMS for different mass ratio through a uniaxial tensile test. g) Stress‐strain curves for pure SEBS, JCMS, and wJCMS through a uniaxial tensile test. Inset shows Young's modulus difference in the initial stage of test. h) Output voltage performance after various mechanical deformations. Bending, rolling, and twisting were carried out 500 times each in order. i) The output voltage performance of the wJCMS measured under strain. Note that we experimented on a 1cm × 1cm effective area to account for the reduction of area under strain.

The SEM images in Figure S9, (Supporting Information) show surfaces at mass ratios of 0, 37.5, 50, 60, and 75 wt.%. At a mass ratio of 60 wt.% in JCMS, the diminishing SEBS exposes CoMOFs on the surface, and at 75 wt.%, CoMOFs are fully exposed. Surface roughness measurements for each mass ratio of JCMS (Figure S10, Supporting Information) reveal that Rq values increase with mass ratios of 0, 50, 60, and 75 wt.%. The Rq values increased following the mass ratios of 0, 50, 60, and 75 wt.%. However, membrane softness begins to decrease beyond 60 wt.% (**Figure** **4**f). In membranes with exposed hard particles, contact occurs only at particle surfaces, reducing the effective contact area. Consequently, despite an increase in the dielectric constant, TENG performance may be diminished due to the reduced contact area.

Another factor influencing the change in the correlation between the dielectric and TENG is the leakage current. Beyond a 60 wt.% mass ratio, the SEBS charge blocking layer is reduced, and the interface between SEBS and CoMOFs layers weakens, as observed in the cross‐sectional SEM images (Figure S6, Supporting Information). Consequently, the weakened interface may diminish the additional charge trapping effect of SEBS's aromatic ring. Charge dissipation test (Figure S11, Supporting Information) shows surface potential decay over time for 0, 50, and 75 wt.%. The decay over time is slowest for 50 wt.% sample, followed by 0 wt.%, and then 75 wt.%. It is notable that the decay overtime for 0 wt.% (SEBS‐only) is slower than that of 75 wt.% JCMS. This suggests that the aromatic rings at SEBS acted as charge‐trapping elements and trap charges at the interface with CoMOFs in JCMS.^[^ ^39^ ^]^ Therefore, in the 75 wt.% sample, which forms an unstable interface due to the decreased amount of SEBS, not only is the decay rate accelerated, but the charge capacity is also reduced, as demonstrated in Figure S8, (Supporting Information). Thus, the absence of a charge blocking layer leads to leakage current, reducing surface charge density despite an increase in dielectric constant, potentially diminishing TENG performance. This aligns with trends observed in previous research.^[^ ^40^ ^]^

Before implementing the JCMS TENG in real‐field applications, its stability was investigated under various environmental temperatures and relative humidities (RH). It is obvious that the stability of JCMS is important in order to get durable TENG performance under open atmosphere. By considering the highest temperature of the environment (56.7°C), the thermal stability of JCMS was evaluated, as shown in Figure S12, (Supporting Information). No observable changes in residual mass of JCMS were found even at temperatures up to 80°C. This supports the thermal stability of JCMS, making it a viable option for utilization in the proposed TENG device in open‐air environments. To comprehend the influence of RH on the output tribo‐electric voltages of JCMS TENG, we meticulously investigated its performance as RH varied from 30% to 70% (Figure S13, Supporting Information) at room temperature. The JCMS TENG has been found to maintain output performance levels above 88% up to 50% RH, but there is a significant reduction in performance beyond that point due to water molecule absorption at the active surface.^[^ ^41^ ^]^ As the proposed rehabilitation application is based on indoor RH, which typically ranges from 30% to 50%, the JCMS TENG can be utilized effectively.

In wearable devices, the key to leveraging TENGs is to generate high output power from low force and frequency inputs common in activities of daily living. We evaluated the optimized sample at various frequencies and forces through contact‐separation mode. The TENG experiments were measured when the charge of the JCMS reached saturation due to triboelectrification after a certain period of contact. The optimal sample showed a peak‐to‐peak voltage of 492 V, an output current of 20.2 µA, and a charge of 40.85 nC at a small force of 1 N (Figure 3f and Figure S14, Supporting Information). Remarkably, this output surpasses the performance of the SEBS under a force of 10 N, which generated a peak‐to‐peak voltage of 286 V, an output current of 12 µA, and a charge of 12.28 nC. In addition, the gradual increase of voltage implies that JCMS retains its elasticity even at the high mass ratio due to the additional SEBS layer, resulting in soft mechanical properties.^[^ ^42^ ^]^ To analyze the variation in the TENG signal in relation to the input frequency, we tested it across a range from 1 to 10 Hz. The output performance shows a modest enhancement as the frequency increased (Figure S15, Supporting Information). The optimal sample showed a peak‐to‐peak voltage of 880 V, an output current of 22.8 µA, and a charge of 56.18 nC at a frequency of 3 Hz. In daily life, most joint movements of the body occur at low frequencies ≈3 Hz^[^ ^43^ ^]^. Therefore, the high operating power at low frequencies of JCMS‐TENG is suitable for human motion sensing. To examine the output of the optimized JCMS‐TENG, we measured the current change for various resistances (Figure 3g). The maximum instantaneous peak power density reached 10.89 W m^−2^ at 100 mΩ with a current of 9.9 µA. Figure 3h shows the comparison of the peak‐to‐peak voltage and peak power densities with MOFs‐based TENGs.^[^ ^31^, ^44^, ^45^, ^46^, ^47^, ^48^, ^49^, ^50^, ^51^ ^]^ This study demonstrates the remarkable values of both peak‐to‐peak voltage and peak power density as compared to those of the previously reported MOFs‐based TENGs. It is mainly attributed to the synergistic effect of the charge trapping and the charge blocking effect of the JCMS‐TENG.

TENGs require at least one electrode to form a path for electrons due to electrostatic induction. Therefore, the inherent insulating properties of polymers can be interfered with in the fabrication of composite‐based TENGs. Typically, stretchable and conductive materials like hydrogel^[^ ^52^ ^]^ and liquid electrode^[^ ^53^, ^54^ ^]^ are used for wearable TENGs. However, the conductivity of these matrix materials limits the charge‐trapping fillers to ensure the continuous entrapment of charges, constraining potential improvements. An alternative approach involves attaching a stretchable electrode by incorporating percolated particles onto a polymer surface.^[^ ^26^ ^]^ These solutions can be bulky, increase tensile stress, or lack stretchability. Hence, it is desirable to develop thin, flexible, and stretchable electrodes that can avoid causing discomfort to the user. Here, we fabricated stretchable silver nanowire (AgNW) wrinkled electrodes on SEBS side of JCMS utilizing the surface dissolution behavior of polymers. The wrinkles on the JCMS were induced by spraying a solution with silver nanowires after 100% pre‐stretching the prepared JCMS (Figure 4a). The solution comprised a homogeneous mixture of toluene, ethanol, and silver nanowires in a certain ratio. Given that toluene can easily and quickly loosen SEBS chains, an instantaneous dissolution occurs on the top surface when the solution is sprayed and contacts the surface of SEBS. The detailed fabrication process to obtain the wJCMS is depicted in Figure S16 (Supporting Information). As depicted in Figure 4b, AgNWs are partially embedded in the surface of SEBS, enhancing the mechanical stability under deformation. To investigate the effect of surface dissolution, the ratio of toluene‐ethanol sprayed solution is adjusted to compare the stability. Figure 4c illustrates the change in resistance with applied strain for the wJCMS fabricated with 100% prestrain and different ratios of the sprayed solution. The volume ratios of ethanol and toluene at 1:0.045 (blue line) and 1:0.09 (red line) show a higher resistance change than 1:0.14 (black line) at 100% strain. It is attributed to the lower toluene ratio, resulting in fewer AgNWs being absorbed by the SEBS surface. This is evident in the cracked surface morphology of the sprayed solution with a toluene concentration of 0%, as shown in Figure S17 (Supporting Information). An increased ratio of the toluene‐ethanol sprayed solution can further enhance the mechanical and electrical properties of the electrode by embedding more AgNWs inside the SEBS. However, it may also penetrate the thin SEBS layer, establishing an electrical connection that inhibits the function of charge blocking layer. As a result, it is desirable to limit the surface dissolution effect to the superficial layer of SEBS, preventing further penetration into the dense CoMOF layer. In the optimized ratio of toluene‐ethanol, as shown in Figure S18 (Supporting Information), the AgNWs do not penetrate into the region where the CoMOFs layer is located; instead, they remain on the surface. A SEM image demonstrating the surface morphology confirms that the percolation of AgNWs remains unbroken at 100% strain (Figure 4d), proving the mechanical robustness of the wrinkled electrode. This wrinkled electrode can be utilized in stretchable TENGs, collaborating with the intrinsic stretchability of JCMS. As previously mentioned, the sedimentation‐assisted membranes demonstrate remarkable stretchability, even with a high loading of filler materials. The digital photograph shows that the JCMS with a 50 wt.% mass ratio is reversible after more than 300% strain due to the excellent stretchability of SEBS (Figure 4e). To quantitatively measure the stretchability of the JCMS, a uniaxial tensile test was conducted for each mass ratio (Figure 4f). As a result, JCMS shows excellent stretchability even at high mass ratios. For pure SEBS, failure occurred at 681% strain, and the maximum strain increased until a mass ratio of 37.5 wt.% was reached. This can be attributed to the physicochemical interactions of CoMOFs and SEBS. When the absolute amount of SEBS decreases (above mass ratio of 42.9 wt.%), the maximum strain begins to decrease due to a weakened network of SEBS.

The optimized mass ratio of 50 wt.% maintained the ductile property of SEBS, which broke at a high maximum strain of 626% (Table S3, Supporting Information). However, when the mass ratio exceeded 60 wt.%, there was a notable weakening of the SEBS network. This weakening led to a significant reduction in maximum strain, dropping to 95.4%. More critically, at mass ratios exceeding 70 wt.%, the material exhibited extreme brittleness, fracturing under minimal deformation. Figure S6 (Supporting Information) illustrates this effect, showing a marked decrease in the thickness of the SEBS layer at 60 wt.% mass ratio, and at 70 wt.%, the layer became virtually indistinguishable. This indicates a reduction in both free and CoMOFs‐bound SEBS, suggesting a shift where filler‐filler interactions became stronger than filler‐polymer interactions.^[^ ^55^ ^]^ This shift in interaction dynamics at elevated filler contents disrupts the continuous polymer network of SEBS, leading to diminished tensile strength and elongation at break. The mechanical properties of the 100% prestrained wJCMS were further evaluated through a uniaxial tensile test (Figure 4g). Although the maximum strain is lower compared to the non‐prestrained JCMS, it still exhibits a considerable range of 475%. Interestingly, Young's modulus of wJCMS reduces due to presence of wrinkled structure^[^ ^56^ ^]^ as shown in the enlargement of the 0‐10% strain range (inset of Figure 4g). This reduction has the potential to mitigate the stress experienced by the user of wearable devices.

In everyday life, wearable devices may encounter various types of mechanical strain. Therefore, maintaining triboelectric properties after different mechanical deformations is crucial for wearable TENGs. The fabrication step of wJCMSs might lead to changes in surface morphology due to the need for prestrain. Figure S19 (Supporting Information) displays the difference in surface morphology between JCMS and wJCMS. The CoMOF remains embedded within the wJCMS even after the JCMS has been prestrained. Figure S20 (Supporting Information) compares the output voltage of the JCMS and wJCMS. The wJCMS shows a peak‐to‐peak voltage of 130 V, a 9% enhancement over the 119 V voltage of the JCMS. To examine the mechanical sustainability, long‐term durability experiment of wJCMS was conducted under an external force of 10 N and at a frequency of 8 Hz in the contact‐separation mode (Figure S21, Supporting Information). The wJCMS demonstrated exceptional robustness, enduring up to 5000 cycles without any performance degradation. This suggests that the wJCMS possesses elastomeric properties due to the support of the SEBS, resulting in no plastic deformation in continuous contact with the PTFE at external forces up to 10 N. The output voltage of the wJCMS‐TENG was also evaluated after various mechanical deformations (Figure 4h). Measurements were taken for one sample through 500 cycles of bending, rolling, and twisting. The peak‐to‐peak voltage after twisting is 212 V, representing a 10.2% reduction from the initial state of 236 V. The output voltages of the wJCMS when stretched were also evaluated with different tensile strains for potential applications in wearable TENGs (Figure 4i). To quantitatively measure the output voltages, the same contact area of 1cm × 1cm was used, accounting for the reduction in width with stretching. Under both fixed‐end conditions, there is an initial performance decline due to the wrinkle that forms when stretching a strip type (i.e., rectangular thin film),^[^ ^57^ ^]^ but then the signal is maintained without degradation up to 100% strain.

**Figure 5:**
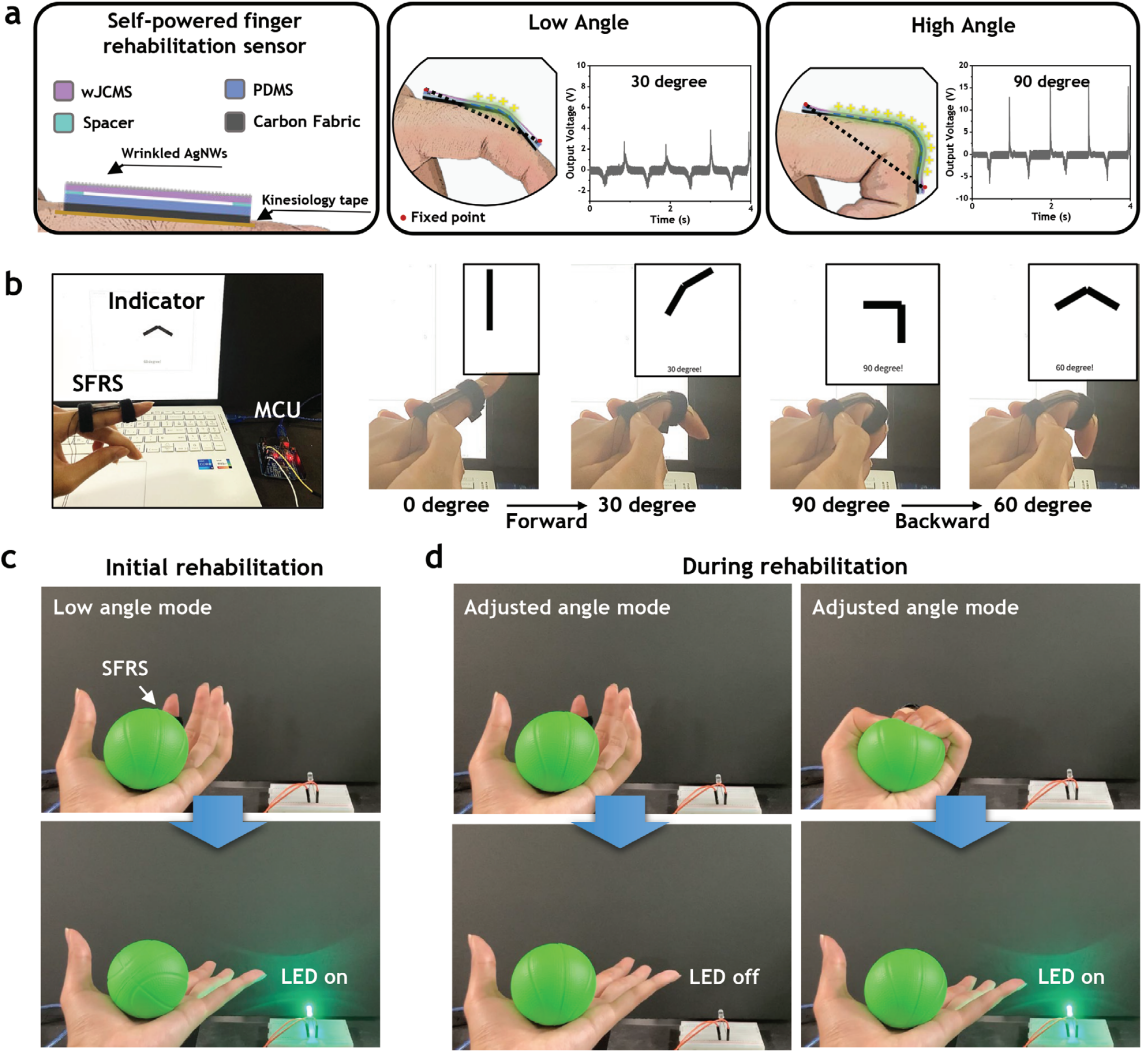
Design and Application of wJCMS‐TENG as a self‐powered finger rehabilitation sensor. a) Design of a self‐powered finger rehabilitation sensor using wJCMS. All components are made of stretchable materials. The contact area varies depending on the angle of the finger bend, which allows the sensor to determine how much the finger is bent. b) The illustration of the SFRS that reacts reversibly to finger angles, integrated with a data storage and display system. Different voltage values for 0°, 30°, 60°, and 90° can be displayed using MCU. c) Finger rehabilitation sensor at the beginning of rehabilitation. LEDs light up on targeted low‐angle finger movements. d) Finger rehabilitation sensor during rehabilitation. By adjusting the target angle, the LED does not light up at low angles and only lights up when the target angle is achieved.

The exceptional mechanical and triboelectric properties of wJCMSs have been utilized to develop a soft TENG angle sensor suitable for applications in self‐rehabilitation healthcare for the fingers. Fingers play a crucial role in our daily routines, enabling us to carry out a range of tasks from fundamental ones such as eating food or picking up objects, to more intricate activities like typing or playing musical instruments. When these significant functions are compromised due to injury, disease, or surgery, finger rehabilitation becomes essential. Self‐rehabilitation offers a potent and convenient method for regaining finger functionality and strength at one's own pace and comfort level. A self‐powered active angle sensor can provide progress feedback by recording and measuring finger movement or angle without additional electrical input to sensor. These sensors can be integrated with smartcare systems to analyze the sensor data, recommend personalized exercises, and provide objectives to improve technique and outcomes. These systems leverage technology to make self‐rehabilitation of fingers more effective, engaging, and customized to individual needs, promising a quicker and smoother recovery journey. For these self‐powered rehabilitation devices, it is essential that the user is not constrained by the rigidity of sensors, and it must respond to small, slow movements and forces. In this context, the high output at low forces and frequencies, high stretchability, and low stiffness of wJCMSs are highly appropriate. **Figure** **5**a presents a self‐powered finger rehabilitation sensor (SFRS) based on wJCMSs. To fabricate SFRS, a PDMS was employed as the negative material, with stretchable carbon fabric used as the electrode. An acrylic tape spacer with a thickness of 3 mm was attached to both ends, with the wJCMS fixed to this spacer. It was affixed to the proximal inter‐phalangeal (PIP) of the index finger using a non‐conductive tapping bandage. When the finger joint moves, the wJCMS constrained at both ends attempts to stay straight like a bow, creating slight tension along the dashed line. Depending on the angle created by the PIP, the contact area of the PDMS and wJCMS changes, altering the voltage signal accordingly. The generated voltage signal (positive value) during detachment is ≈3 V at 30° and 16 V at 90°. As shown in Figure S22 (Supporting Information), the output voltage can be differentiated by the operating frequency. Accordingly, SFRS enables to monitor the output value of a sensor with a different voltage depending on the angle through the microcontroller (MCU). It should be noted that the maximum voltage input for the Arduino Uno is 5 V. Therefore, a voltage divider was employed, and the corresponding signal is displayed in Video S1 (Supporting Information). Through serial communication with the MCU, the all‐stretchable SFRS successfully differentiated and displayed between 30, 60, and 90° (Figure 5b; Video S2, Supporting Information). Based on the stored data, the health center can assess the patient's finger rehabilitation status. It is worth noting that the SFRS possesses an all‐stretchable feature, allowing it to sense angles reversibly, as demonstrated in Figure 5a. Conversely, present flexible TENG‐based self‐powered sensors mainly rely on flexibility for detecting human motion. Such sensors tend to be temporarily fixed at high angles, ultimately reducing their capacity to detect lower angles.^[^ ^58^, ^59^ ^]^ Therefore, this reversible sensing capability of the SFRS is advantageous for accurately monitoring rehabilitation progress. A comparison of the characteristics of SFRS with other wearable TENG‐based self‐powered sensors is provided in Table S4 (Supporting Information).

The measured angle data will be reported to the medical center, enabling it to gauge the patient's rehabilitation status accurately. Based on the patient's progress, the medical center can then adjust the goals for the range of motion in the fingers during rehabilitation. This adjustment allows patients to evaluate their performance (Figure 1d). Each patient can easily monitor their rehabilitation progress through LED lights, thereby making finger rehabilitation more effective and smoother. Figure 5c shows a low voltage threshold applied at the onset of rehabilitation, which triggers the LED even at a small angle. Figure 5d illustrates the situation during rehabilitation, where a higher voltage threshold is applied. Here, the LED does not illuminate at low angles and only lights up when a certain angle is reached, enabling self‐counting for patients. These operations are also displayed in Video S3, (Supporting Information). The fabricated wJCMS is not only self‐powered by exploiting its stretchable characteristics, but also exhibits excellent performance in charging capacitors or powering LEDs (Figures S23 and S24, Supporting Information), showing the potential usage for energy harvesting system. This current work demonstrates the potential of stretchable sensors in smart healthcare as angle sensors for rehabilitation. Beyond rehabilitation, these devices also hold potential as guidance tools for everyday exercise routines, integrating with existing exercise equipment or systems. The wJCMS exhibits great potential as a wearable TENG due to its simple fabrication method, low stiffness, and high signal generation facilitated by the utilization of the JCMS membrane.

## 3 Conclusion

In this study, we have developed a Janus CoMOF‐SEBS membrane with charge trapping and blocking bifunctionality, suitable for self‐powered wearable sensors requiring stretchability, flexibility, and high‐power output. The bifunctional JCMS membrane was obtained through the use of unique gravitational sedimentation techniques. The resulting JCMS demonstrated both the charge trapping and charge blocking capabilities, along with mechanical resilience, flexibility, and superior output performance. The performance was observed to be dependent on the thickness of its charge trapping and blocking layers when utilizing JCMS as a tribo‐positive material in a contact‐separation mode TENG. At a frequency of 5 Hz and an external force of 10 N, the optimized JCMS‐TENG achieved high output values, including a peak‐to‐peak voltage of 936 V, output current of 42.8 µA, and power density of 10.89 W m^−2^, respectively, surpasses the performance of previous MOF‐based TENGs. This improvement was primarily a result of the effects of the specially crafted asymmetric charge trapping and blocking layers. In addition, the resulting JCMS‐TENG displays significant potential for integration into finger rehabilitation devices due to its low Young's modulus, mechanical robustness, and high stretchability. It has the ability to effectively serve as a self‐powered sensor for finger rehabilitation alongside an intelligent healthcare system. This study demonstrates that JCMS has high potential in developing efficient, versatile, and user‐friendly TENGs for energy harvesting systems, wearable devices, and self‐powered sensors.

## 4 Experimental Section

### Preparation of CoMOFs

In this study, CoMOFs are synthesized via conventional solvothermal treatment using the autoclave. The precursors were prepared by mixing cobalt(II) nitrate hexahydrate (Sigma–Aldrich), benzophenone‐3,3′,4,4′‐tetracarboxylic acid dihydrate (BPTC, Sigma‐Aldrich), and N,N‐dimethylformamide (DMF, DAEJUNG, Korea) in a molar ratio of 1:1:300. Then mixture was placed in a Teflon liner and sonicated (POWER Sonic 510) for 30 min for solubilization. The liner was then sealed in an autoclave and placed in the oven at 110 °C for 48 h. The CoMOF particles were collected by filtration and rinsed three to four times with DMF to remove unreacted precursors. The collected CoMOF powder was dried in a vacuum oven at 110°C for 8 h to remove solvent and stored in a vacuum chamber to prevent further reaction.

### Preparation of JCMS

First, the CoMOFs were mixed with 1.7 mL of 1,4‐dioxane (Sigma–Aldrich) and sonicated (POWER Sonic 510) for 1 h. A Styrene‐Ethylene‐Butylene‐Styrene (SEBS, Asahi Kasei) solution diluted in toluene to 80 mg mL^−1^ was then added to the CoMOFs suspension and mixed for 1 min using a magnetic stirrer at 300 rpm to induce a homogeneous phase. The ratio of 1,4‐dioxane to toluene was fixed at 1:3 volume ratio. Next, it was cast into a 5.5 cm diameter glass mold immediately after stirring to prevent further sedimentation. The cast suspension was dried in an oven at 90°C for 1 h after waiting 30 min to induce sufficient gravitational sedimentation. To prevent the solution from tilting to one side during the drying process, the bottom of drying oven was counter balanced using a flat plate with adjustable tilt. The membranes were separated from glass mold after drying using ethanol treatment. The detailed quantity used for each mass ratio of JCMS is summarized in Table S2 (Supporting Information).

### Preparation of wJCMS

To induce surface dissolution, the spraying solution was prepared by adding toluene in a 1:0.14 ethanol‐toluene volume ratio to a solution of silver nanowires dispersed in ethanol. To induce the wrinkled structure, the JCMSs were stretched to 100% and fixed on the glass substrate. To reduce the island effect, the stretched JCMSs were hydrophilized by air plasma treatment (COVANCE‐1MPR, Femto Science) for 5 min. Next, the SEBS surface of JCMSs was sprayed using conventional air spray gun. The distance between the membrane and the spray gun was kept at 15 cm, and the solution was sprayed ten times in the longitudinal direction. The membrane was then returned to its original length. The fabricated wJCMS were stored in an oven at 50°C for 15 min and then stored in a vacuum chamber until use.

### Material Characterization

The characterization of JCMS and wJCMS was carried out through a comprehensive set of analyses. Field Emission Scanning Electron Microscopy (FE‐SEM) images were obtained using a HITACHI SEM (SU5000). To explore the elemental composition of wJCMS, the Energy‐dispersive X‐ray spectroscopy (EDS) of the SEM was employed. Transmission Electron Microscopy (TEM) analysis and corresponding elemental mapping of the CoMOFs were performed using a Talos F200X (FEI) with a STEM‐High Angle Annular Dark Field attachment. Fourier Transform Infrared Spectroscopy (FTIR) was recorded using a Nicolet iS50 instrument (Thermo Fisher Scientific). The CoMOF was analyzed in the form of KBr pellets, while attenuated total reflectance (ATR) signals were used for SEBS and JCMS. In‐Situ X‐ray Photoelectron Spectroscopy (XPS) was performed using Thermo Fisher Scientific K‐Alpha. Ar etching was conducted for 480 s to obtain clear information about the cobalt component. The mechanical properties were examined through uni‐axial tensile tests using a universal testing machine (AGS‐X series, Shimadzu) at an elongation rate of 20 mm min^−1^. For these tests, each sample was prepared into a dog‐bone specimen of 4 mm thickness and a gauge length of 25.5 mm facilitated by a custom‐made punching machine. A Precision LCR Meter (E4980AL, Keysight) and a dielectric test fixture (16451B, Keysight) were used to measure the permittivity for each mass ratio of JCMS at room temperature. To eliminate the noise caused by the formation of an air gap between the test fixture and the sample, platinum was sputtered through a 2 mm thick ring‐shaped mask on the JCMS. The dielectric constant ε_*r*_ was calculated using the following equation:

(3) $$\;\varepsilon_{r}=\frac{C_{p}\times t_{a}}{A\times \varepsilon_{o}}\;$$

where *C_p_* is the equivalent parallel capacitance, *t_a_* is the average thickness of the JCMS, *A* is the area of the thin film electrode, and ε_*o*_ is the vacuum permittivity (8.854 × 10^−12^ F m^−1^). The surface potential and decay of JCMS were measured using an electrostatic field meter (FMX‐004, SIMCO) at 18°C and 43% RH. The surface roughness was measured at room temperature using an atomic force microscope (FX‐40, Park systems).

### Fabrication of TENGs

Commercial PTFE (80 µm thickness) is used as the negative triboelectric material, while either JCMS or wJCMS was utilized as positive triboelectric layer. Aluminum tapes were used as electrodes for both JCMS and PTFE. A 3 × 3 cm acrylic plate (2 mm thickness) is used as substrate for JCMS‐TENG measurement. A 1.5 × 1.5 cm acrylic plate is used for wJCMS‐TENG measurement, unless otherwise noted.

### Triboelectric Output Measurements

A mechanical shaker (S510575, TIRA) was connected to a function generator (AFG 3022, Tektronix) and power amplifier (BAA 120, TIRA) to generate a step function input. An oscilloscope (DPO 3052, Tektronix, USA) and a voltage probe (P5100A, Tektronix) were used to measure the output voltage and current. In addition, a low‐noise current amplifier (SR570, Stanford Research Systems) was used to measure the output current, minimizing noise interference. A force sensor (1051V2, Dytran) was attached to the top of the shaker to measure the force between the shaker and substrate. A photographic description of the experimental setup is presented in Figure S25 (Supporting Information). The instantaneous peak power density was calculated using the following equation:

(4) $$\;P_{\mathrm{instantaneous}}=\frac{I^{2}R}{A}\;$$

Here, *P*_instantaneous_ represents the instantaneous power density, *I* is the positive output current for each resistance, *R* is the value of the connected resistance, and *A* is the area of the friction surface. The current was measured by serially connecting each resistance with a current amplifier, as shown in Figure S26 (Supporting Information). All TENG experiments were conducted at 18–25° and 40%−45% relative humidity.

### Self‐Rehabilitation Sensor System

To fabricate the stretchable self‐rehabilitation sensor system, all components were made from stretchable materials. The PDMS forming the tribonegative layer was made by mixing the base and curing agent in a ratio of 20:1, followed by curing process in a 60°C oven for 30°min. The electrode for the PDMS was made of stretchable carbon cloth. For the tribopositive layer and electrodes, wJCMS with dimensions of 1 × 3 cm was used. Kinesiology tape was attached to the PIP for insulation. A 3 mm thick acrylic tape was used to attach the wJCMS to the ends of the carbon cloth and PDMS. Velcro was then secured to both ends to facilitate wearing. Each electrode was connected to an Arduino UNO to transmit the electrical signal and activate the LED.

## Conflict of Interest

The authors declare no conflict of interest.

## Supporting information

Supporting Information

Supplemental Video 1

Supplemental Video 2

Supplemental Video 3

## Data Availability

The data that support the findings of this study are available from the corresponding author upon reasonable request.
