# Supplementary material for: Janus CoMOF‐SEBS Membrane for Bifunctional Dielectric Layer in Triboelectric Nanogenerators
Source: Adv Sci (Weinh). 2024 Jan 29;11(14):2307656. doi: 10.1002/advs.202307656 (PMC11005725; doi:10.1002/advs.202307656)
Supplement: Supplementary file 1 — Supporting Information [file ADVS-11-2307656-s001.pdf]

ADVANCED SCIENCE
Open Access

Supporting Information

for *Adv. Sci.*, DOI 10.1002/advs.202307656

Janus CoMOF-SEBS Membrane for Bifunctional Dielectric Layer in Triboelectric Nanogenerators

*Hyunjoon Yoo, Manmatha Mahato, Ji-Seok Kim, Saewoong Oh, Mousumi Garai, Van Hiep Nguyen, Ashhad Kamal Taseer, Myung-Joon Lee and Il-Kwon Oh**

Supporting Information

Janus CoMOF-SEBS Membrane for Bifunctional Dielectric Layer in Triboelectric Nanogenerators

Hyunjoon Yoo §, *Manmatha Mahato* §, *Ji-Seok Kim, Saewoong Oh, Mousumi Garai, Van Hiep Nguyen, Ashhad Kamal Taseer, Myung-Joon Lee and Il-Kwon Oh* *

H. Yoo, M. Mahato, J.-S. Kim, S. Oh, M. Garai, V. H. Nguyen, A.K. Taseer, M.-J. Lee and I. K. Oh

National Creative Research Initiative for Functionally Antagonistic Nano-Engineering, Department of Mechanical Engineering, Korea Advanced Institute of Science and Technology (KAIST), 291 Daehak-ro, Yuseong-gu, Daejeon 34141, Republic of Korea.

* Correspondence and requests for materials should be addressed to I.-K. Oh (email: ikoh@kaist.ac.kr).

§ These authors contributed equally to this work

Keywords: stretchable TENG; metal-organic frameworks; self-rehabilitation; bifunctionality; gravitational sedimentation

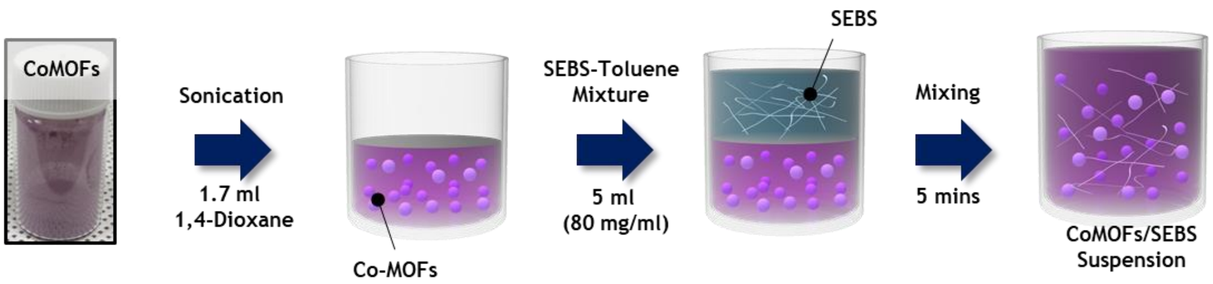

Figure S1. Schematic illustration of the preparation process for CoMOFs/SEBS suspension.

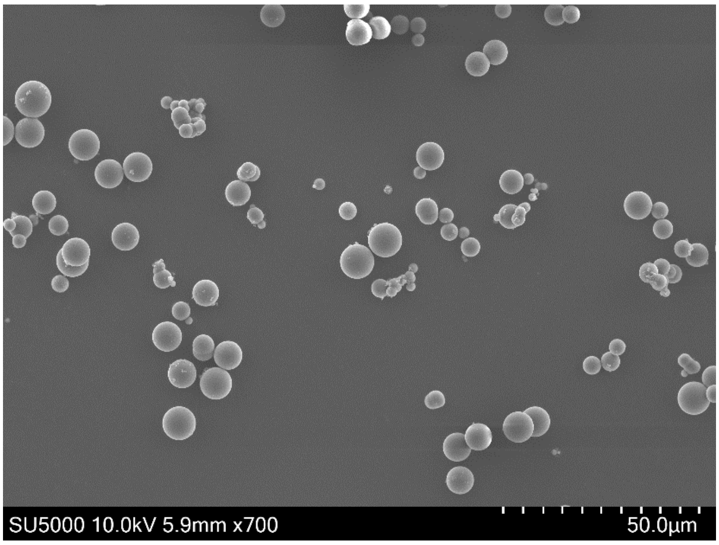

Figure S2. SEM image of spherical CoMOFs particles, grown by solvothermal treatment, with diameters of 1-10 micrometers.

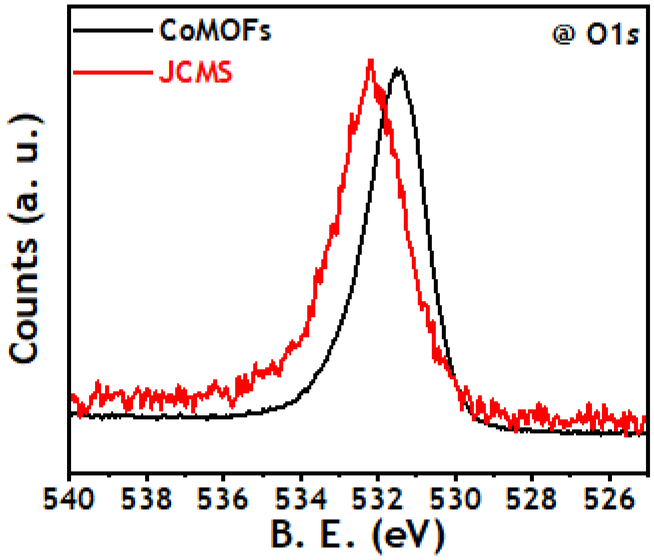

Figure S3. XPS spectra of CoMOFs and JCMS centered on O 1s.

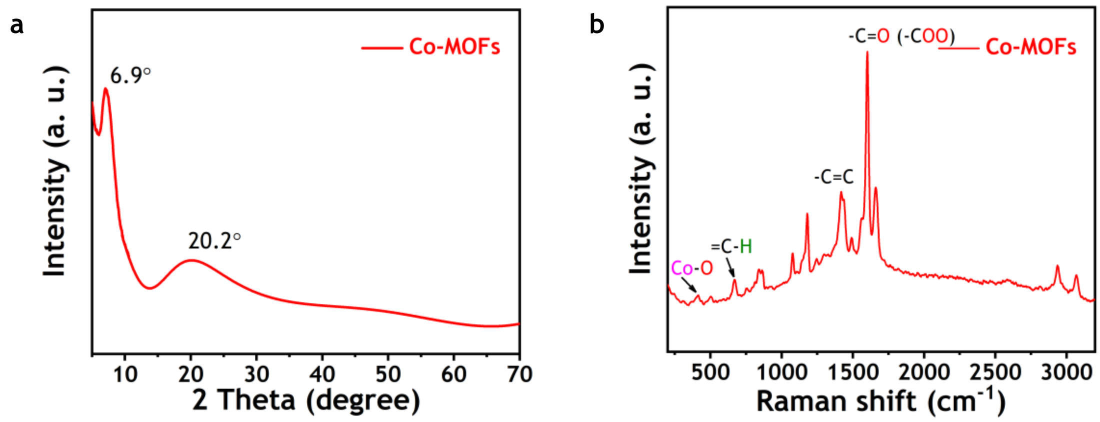

Figure S4. Structural characterization of Co-MOFs. (a) XRD and (b) Raman spectra of Co-MOFs active materials.

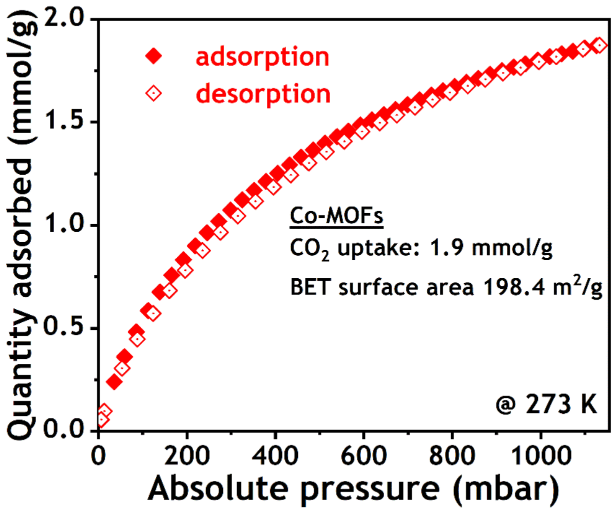

Figure S5. Carbon-di-oxide (CO_2) adsorption-desorption isotherm of Co-MOFs at 273 K.

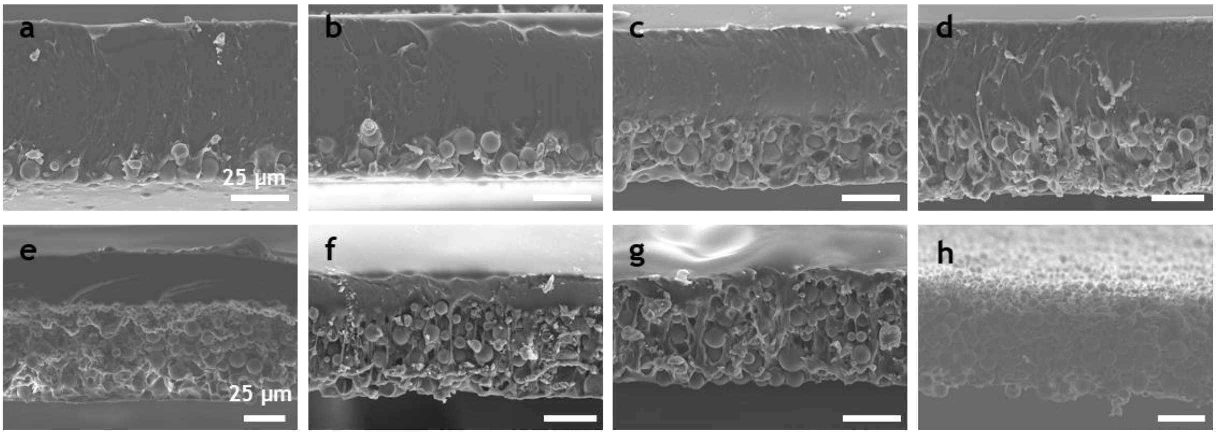

Figure S6. Cross-sectional SEM images of JCMSs at different mass ratios. a-d) The quantity of SEBS was fixed, and the amount of CoMOFs was adjusted (9.1 wt% to 37.5 wt%). The

thickness of the CoMOFs dense layer increases as the mass ratio increases. e-h) The quantity of CoMOFs is fixed and the amount of SEBS is adjusted. As the mass ratio increases, the thickness of the pure SEBS layer gradually decreases.

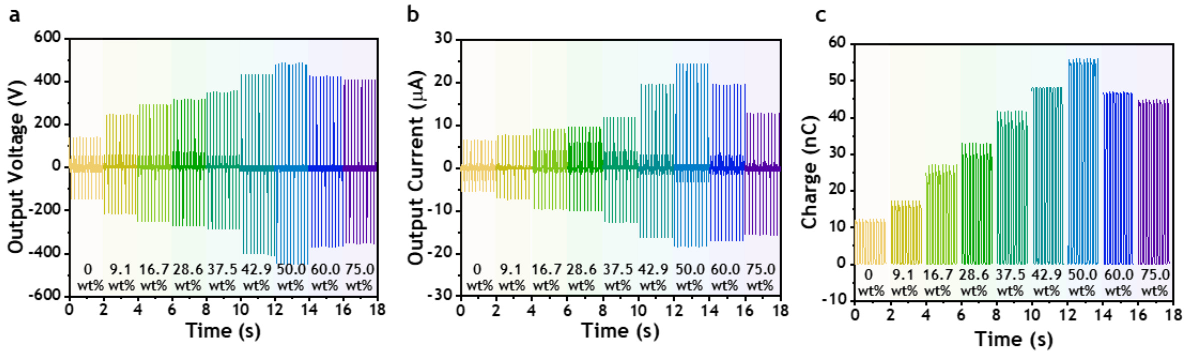

Figure S7. The output performance of JCMS-TENG with different mass ratio. (a) Output voltage, (b) current, and (c) charge.

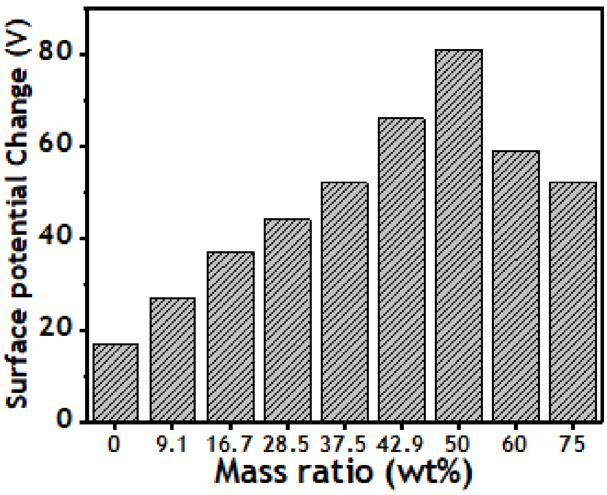

Figure S8. Surface potential change of JCMS depending on the mass ratio.

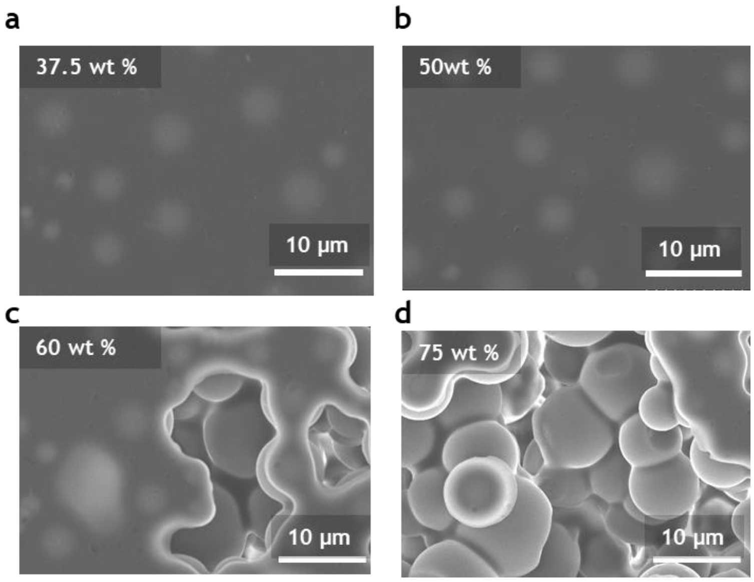

Figure S9. Surface SEM images of the dense area of CoMOFs at each mass ratio.

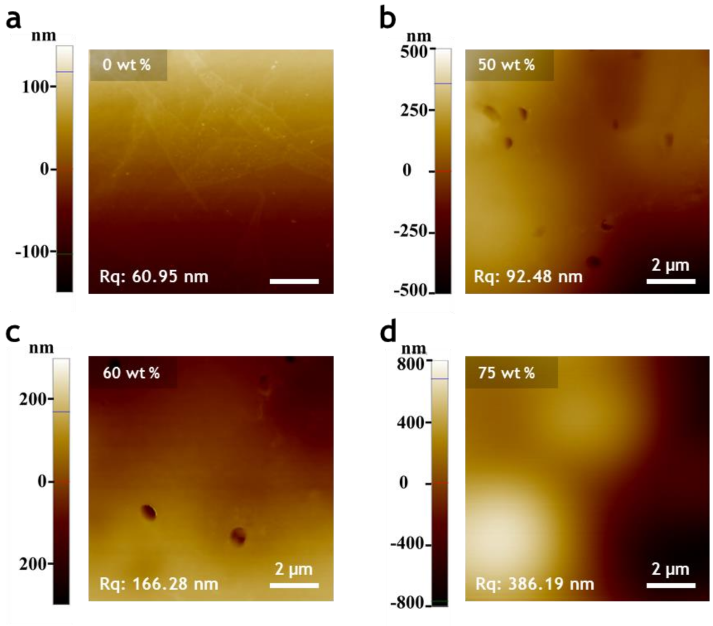

Figure S10. AFM images and Rq values of the dense area of CoMOFs at each mass ratio.

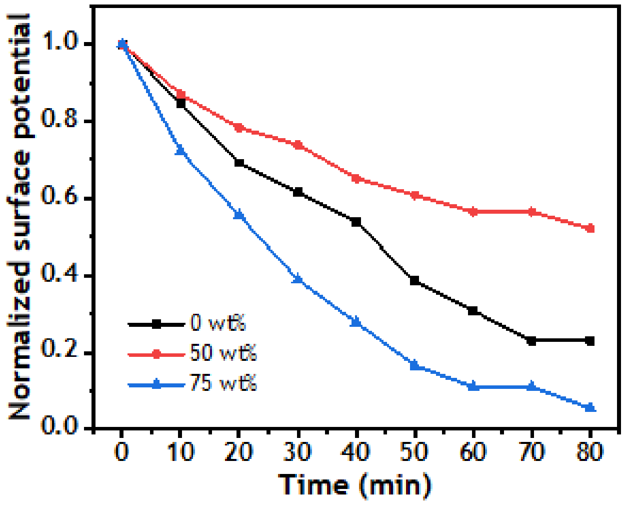

Figure S11. Normalized surface potential decay of JCMS over time.

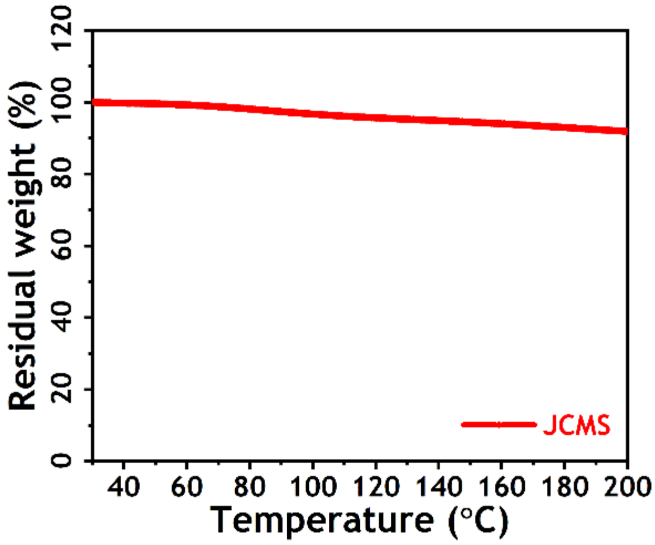

Figure S12. Thermogravimetric (TG) analysis of JCMS.

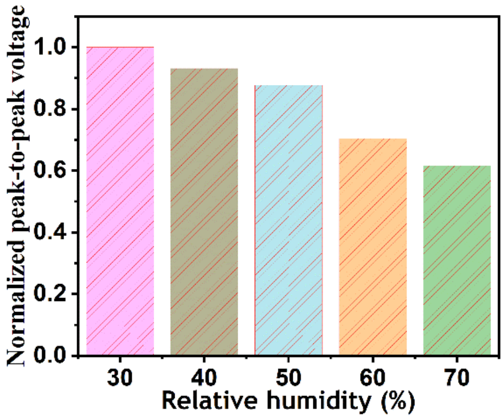

Figure S13. Output performance of JCMS TENG with the change in relative humidity.

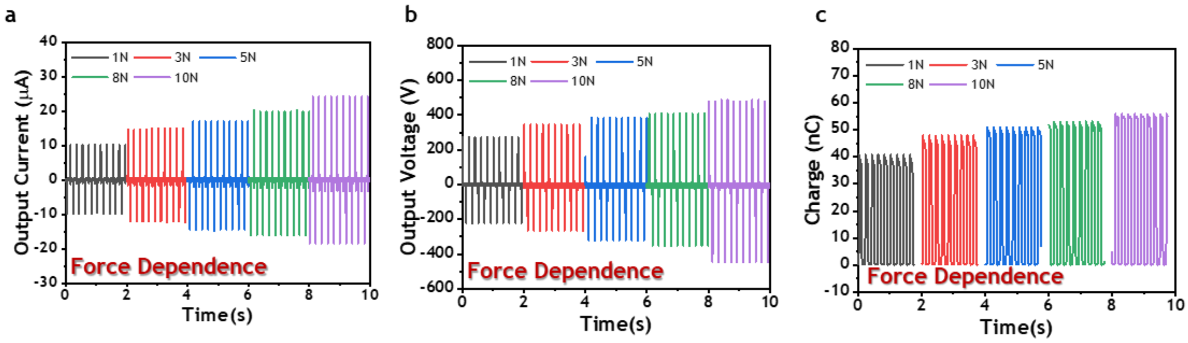

Figure S14. The output performance of optimized JCMS-TENG under various external forces. (a) Output current, (b) voltage, and (c) charge.

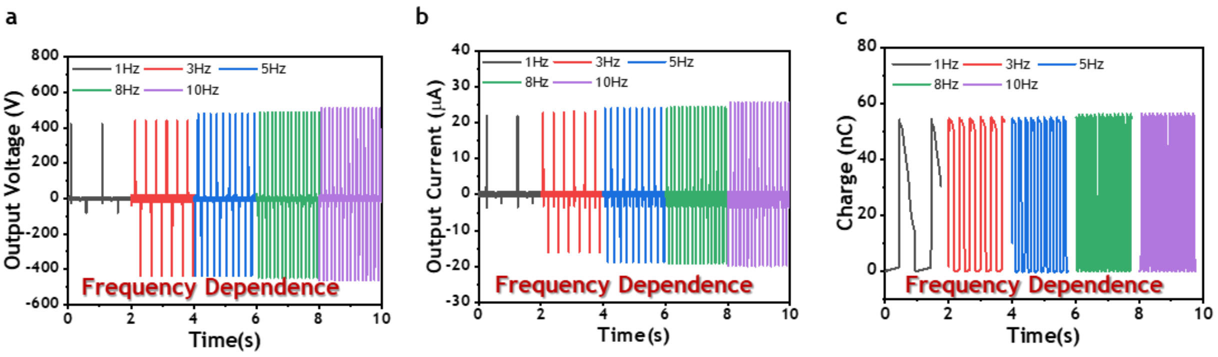

Figure S15. The output performance of optimized JCMS-TENG at various frequencies. (a) Output voltage, (b) current, and (c) charge.

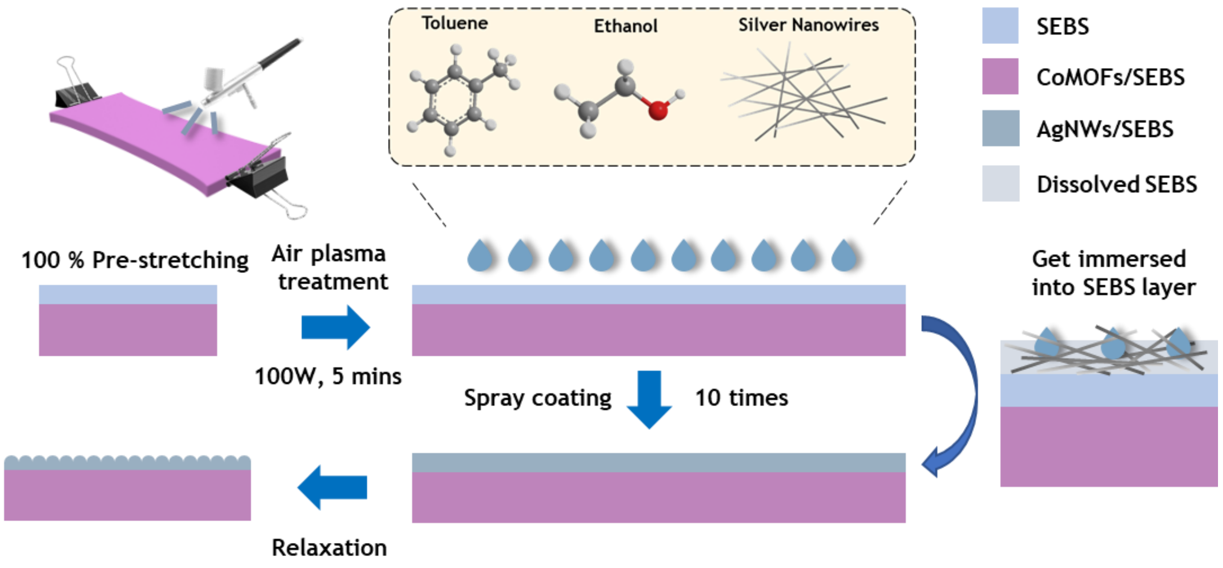

Figure S16. Schematic of wJCMS fabrication using surface dissolution effect.

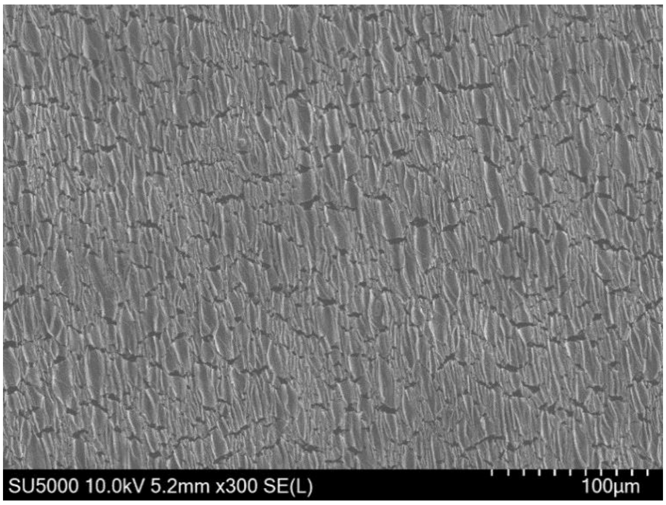

Figure S17. SEM image of wJCMS with AgNWs using an ethanol-based solution. The fabrication process is identical to the sample preparation method that utilizes the surface dissolution effect.

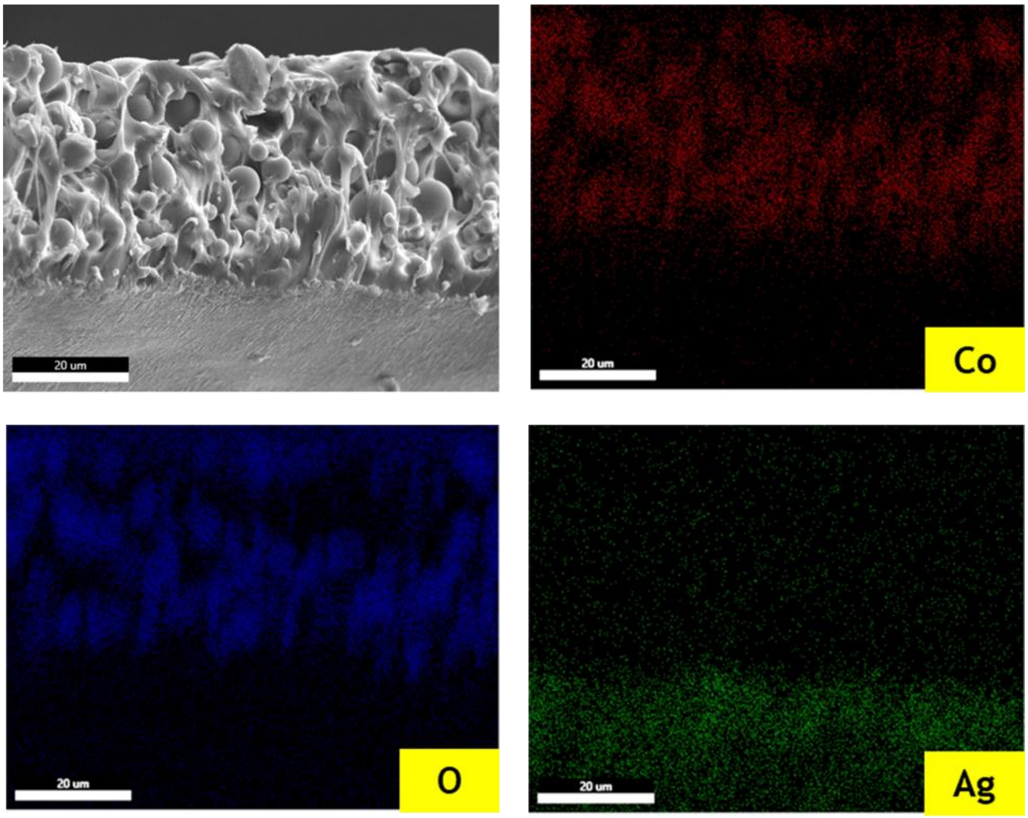

Figure S18. EDS images of wJCMS, displaying presence of CoMOFs and partially embedded AgNWs.

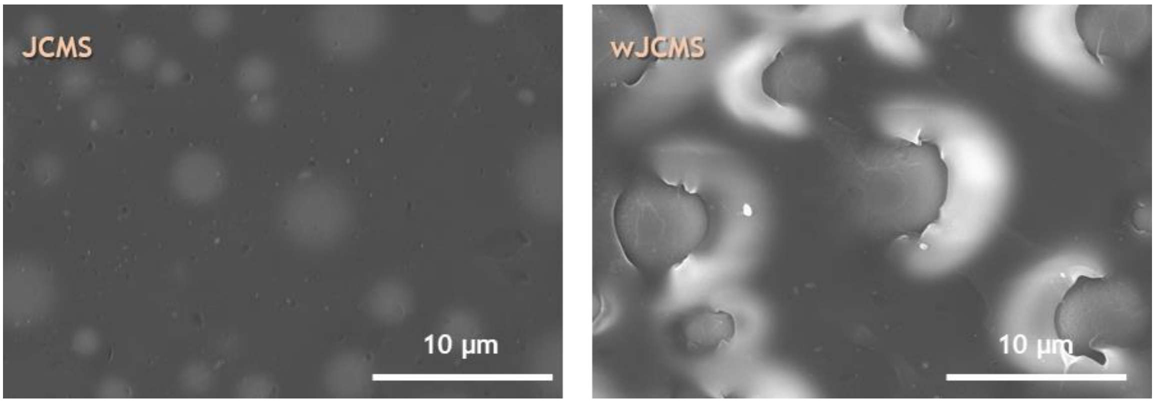

Figure S19. SEM images demonstrating change of surface morphology on CoMOFs dense layer side of wJCMS resulted by 100% pre-strain of JCMS.

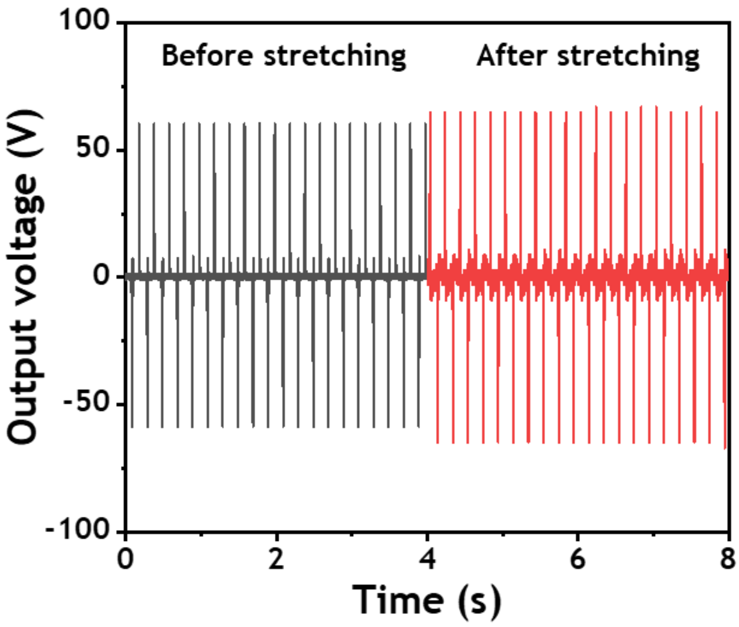

Figure S20. Change in output voltage before and after stretching the JCMS at 100% strain. The identical sample was temporarily attached to a 1cm x 1cm acrylic plate and driven by contact-separation at a force of 10 N and a frequency of 5 Hz.

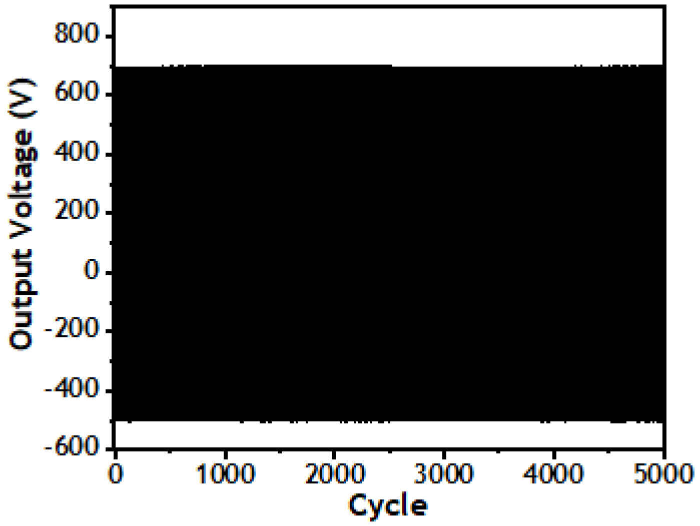

Figure S21. Long-term cycle test of wJCMS. The JCMS was attached to a 3cm x 3cm acrylic plate and driven by contact-separation at a force of 10 N and a frequency of 5 Hz.

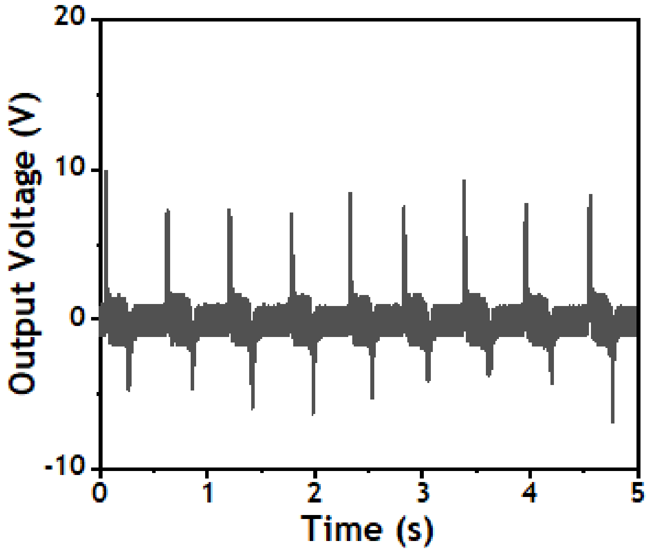

Figure S22. The output voltage of the self-powered finger rehabilitation sensor during fast finger movement.

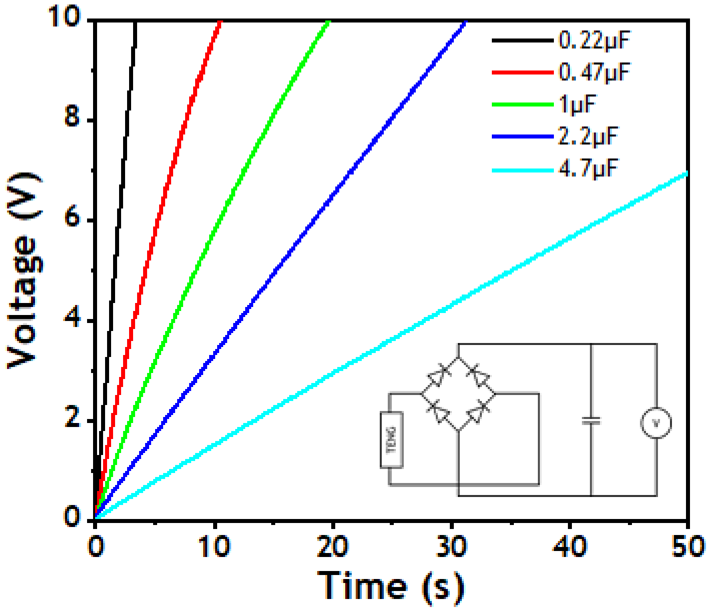

Figure S23. The **c**harging process for different capacitors. A corresponding circuit diagram is provided in the inset. wJCMSs-TENG were driven by a contact-separation under a force of 10 N and at a frequency of 5 Hz.

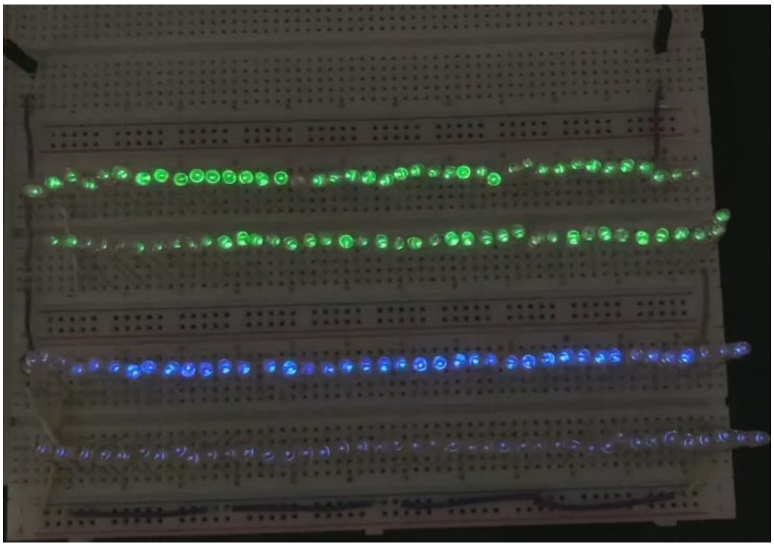

Figure S24. Digital photography driving over 160 LEDs. wJCMSs-TENG were driven by a contact-separation under a force of 10 N and at a frequency of 5 Hz.

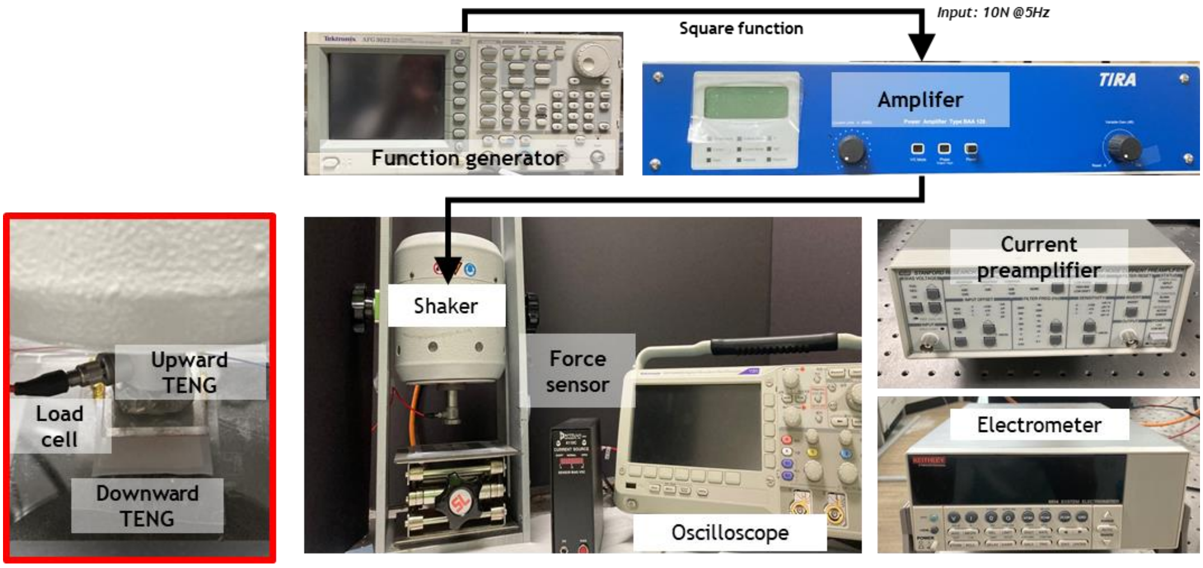

Figure S25. Experimental set-up for examining the TENG performances. All experiments were driven by a contact-separation with a force of 10 N and a frequency of 5 Hz, unless otherwise noted.

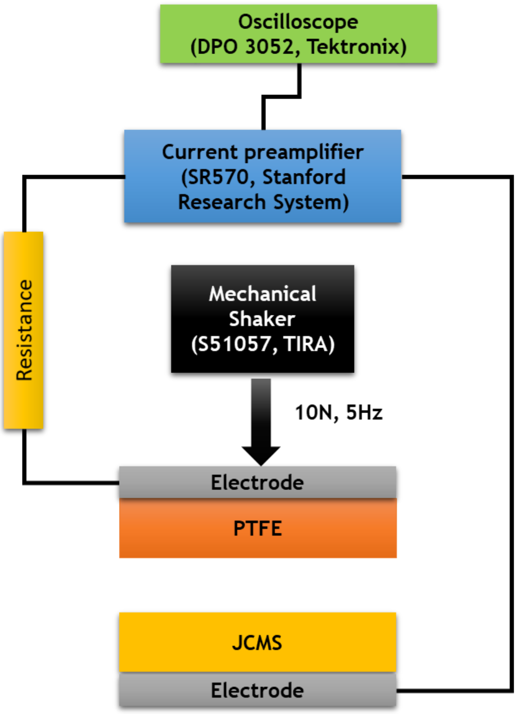

Figure S26. Schematic diagram of the circuit for measuring instantaneous power density.

Table S1. Advantageous features of JCMS TENG over other TENGs based on Janus structures.

**Ref.**	**Device configuration**		**Application**				**Performance**		
	Positive material	Negative material	Electrode (Positive/Negative)	Application	Operating mode	Flexibility	Stretchability/Elongation at break	Output Voltage @ Optimal mass ratio	Output Voltage Increment
[2]	Zn/Co MOFs+PVDF	PVDF	Cu/Cu	-	Contact-separation mode (two electrodes)	-	-/-	623.3 V@ 50wt%	163.6%
[3]	Janus nanobelt array [PANI/CNTs/PMMA]//[Tb(BA)3phen/PMMA]	PVDF/PVP nanofibers membrane	Cu/Cu	-	Contact-separation mode (one eletrode)	O	-/-	155V @ 5wt% of PANI to PMMA	166.7%
**This work**	JCMS (CoMOFs +SEBS)	PDMS	Wrinkled AgNW/ conductive carbon fabric	Rehabilitation self-powered sensor	Contact-separation mode (two electrodes)	O	O /475%	936 V@ 50wt%	327%

Table S2. The quantity of SEBS and CoMOFs used in each mass ratio of JCMS.

	**Controlling the quantity of CoMOFs**			
**Mass ratio (wt%)**	**9.1**	**16.7**	**28.6**	**37.5**
**SEBS (mg)**	200	200	200	200
**CoMOFs (mg)**	20	40	80	120
	**Controlling the quantity of SEBS**			
**Mass ratio (wt%)**	**42.9**	**50.0**	**60.0**	**75.0**
**SEBS (mg)**	160	120	80	40
**CoMOFs (mg)**	120	120	120	120

Table S3. Mechanical properties of JCMS according to each mass ratio.

**Mass ratio of JCMS**	**Strain at Break (%)**	**Tensile strength (MPa)**
0 wt%	681.4	16.7
9.1 wt%	821.8	26.4
16.7 wt%	797.3	22.1
28.6 wt%	742.1	17.5
37.5 wt%	720.7	17.2
42.9 wt%	641.8	8.7
50.0 wt%	625.9	7.2
60.0 wt%	95.4	1.63

Table S4. Comparison of prior studies on TENGs based on flexible or stretchable materials as self-powered sensors.

Ref.	Device configuration			Self-powered sensor			
	Positive material	Negative material	Electrode (Positive/Negative)	Application	Operating mode	Flexibility	Strectchability/ Elongation at break
**[3]**	PEDOT:PSS	PUA	PEDOT:PSS/AgNW on PDMS	Self-powered display	Contact-separation mode (two electrodes)	O	-/-
**[4]**	Al	PDMS/CNT	Al/PDMS/CNT	Self-powered physical activity sensor on the leg	Contact-separation mode (one eletrode)	O	O/-
**[5]***	Skin	PP	Skin /Hydrogel electrode	wearable self-powered sensing devices on finger	Contact-separation mode (one eletrode)	O	O/-
**[6]**	ITO on PET	MXene	ITO on PET	Self-powered sensor on the thumb	Contact-separation mode (two electrodes)	O	-/-
**[7]**	Au embedded in PDMS	PDMS	Au embedded in PDMS	Self-powered human-motion detector on finger, knuckle, and wrist	Contact-separation mode (two electrodes)	O	O/-
**[8]***	Skin	PVA/MXene	Skin/-	Self-powered human-motion detector on finger, wrist, knee and throat	Contact-separation mode (one eletrode)	O	-/-
**This work ****	JCMS	PDMS	Wrinkled sliver nanowire/ conductive carbon fabric	Rehabilitation self-powered sensor	Contact-separation mode (two electrodes)	O	O/475%
***Angle detection is available.<br>**Reversible sensing of angular change**							

References

[1] Y. Xie, Q. Ma, H. Qi, X. Liu, X. Chen, Y. Jin, D. Li, W. Yu, X. Dong, *Nanoscale* **2021**, *13*, 19144.
[2] C. Huang, G. Lu, N. Qin, Z. Shao, D. Zhang, C. Soutis, Y.-Y. Zhang, L. Mi, H. Hou, *ACS Appl. Mater. Interfaces* **2022**, *14*, 16424.
[3] M. Qu, L. Shen, J. Wang, N. Zhang, Y. Pang, Y. Wu, J. Ge, L. Peng, J. Yang, J. He, *ACS Appl. Nano Mater.* **2022**, *5*, 9840.
[4] B.-Y. Lee, S.-U. Kim, S. Kang, S.-D. Lee, *Nano Energy* **2018**, *53*, 152.
[5] M.-K. Kim, M.-S. Kim, H.-B. Kwon, S.-E. Jo, Y.-J. Kim, *RSC Adv.* **2017**, *7*, 48368.
[6] Y. Dong, S. S. K. Mallineni, K. Maleski, H. Behlow, V. N. Mochalin, A. M. Rao, Y. Gogotsi, R. Podila, *Nano Energy* **2018**, *44*, 103.
[7] G.-H. Lim, S. S. Kwak, N. Kwon, T. Kim, H. Kim, S. M. Kim, S.-W. Kim, B. Lim, *Nano Energy* **2017**, *42*, 300.
[8] C. Jiang, C. Wu, X. Li, Y. Yao, L. Lan, F. Zhao, Z. Ye, Y. Ying, J. Ping, *Nano Energy* **2019**, *59*, 268.
